# Rainfall alters network structure, while fragmentation results in the breakdown of a mixed-species group of birds

**DOI:** 10.1007/s00442-026-05869-7

**Published:** 2026-02-07

**Authors:** Laura Gómez-Murillo, Jeferson Vizentin-Bugoni, Andrés F. Ramírez-Mejía, Corey E. Tarwater

**Affiliations:** 1 Department of Zoology and Physiology, University of Wyoming, 1000 E. University Ave., Laramie, WY 82071 USA; 2 Programa de Pós-Graduação em Biodiversidade Animal, Departamento de Ecologia, Zoologia e Genética, Instituto de Biologia, Universidade Federal de Pelotas - UFPEL, Campus Capão do Leão, Capão do Leão, RS 96010-900 Brazil; 3 Smithsonian Tropical Research Institute, Apartado 0843-03092, Balboa, Republic of Panama

**Keywords:** Ant-following birds, Army ants, Mixed-species flock, Neotropics, Network dissimilarity

## Abstract

Understanding the influence of species interactions on community structure and biodiversity is a long-standing goal in ecology. While species interactions are predicted to be vulnerable to environmental change, how the environment influences species interactions is not well understood. We used a network analysis approach to examine how network structure and dissimilarity of one type of mixed-species animal group, army ant-following birds, varied along rainfall and habitat suitability (i.e., fragmentation) gradients in Panama. Network structure varied across both gradients and there was high interaction dissimilarity across networks, owing to both species turnover and rewiring. These results highlight the role of the environment in structuring species interactions. Importantly, there was total dissolution of ant-following groups at sites with lower habitat suitability. In drier sites, networks were more speciose, cohesive, and well-connected compared to wetter forests, potentially because of the increased benefits of attending swarms in drier sites. The high cohesion and connections found suggest that ant-following birds may be particularly vulnerable to the loss of important species and environmental change. These ant swarms are an important, shared food resource that draws together a diverse and tightly connected group of birds. Understanding the relationships between the environment and species interactions contributes to our ability to predict how species interactions will change in the future, with cascading effects on community structure and biodiversity.

**Supplementary Information:**

The online version contains supplementary material available at 10.1007/s00442-026-05869-7.

## Introduction

Changes in the environment alter population distribution and abundance, community composition, organismal physiology, and ecosystem function (Sala et al. 2000; Hansen et al. 2001; Asner et al. 2010; Tylianakis and Soper 2013). Owing to the potential for mismatches in phenology, behavior, and abundances between species, species interactions are predicted to be highly sensitive to environmental changes (Parmesan 2006; Tylianakis et al. 2008; Gornish and Tylianakis 2013; Ockendon et al. 2014). The challenges of studying multiple species across time or environmental gradients, however, have hampered understanding of how species interactions are changing now and may change in the future (Parmesan 2006; Bascompte 2010). Network theory provides a powerful tool for quantifying variation in species interactions by providing metrics that reflect community structure and function which are comparable across sites and studies (Krause et al. 2009; Mokross et al. 2014; Farine and Whitehead 2015). Here, we use network theory to examine alterations in species interactions across environmental gradients in Panama.

We examined ecological networks across environmental gradients using one type of species interaction—mixed-species animal groups (MSAGs). MSAGs are common across taxa and are defined as a group of independently moving animals from more than one species, found in close proximity, and interacting with one another (Goodale et al. 2017a, b). The costs and benefits of participating in MSAGs and the presence of key species (important for recruitment and group cohesion) are predicted to alter both the species present and the stability of the interactions (Goodale et al. 2020). For example, MSAGs across elevational gradients in the tropics were composed of a stable core group of species and a more dynamic component of attending species (Muñoz & Jankowski 2016); however, the frequency and the structure of the interactions varied across these different gradients (Tylianakis and Morris 2016; Montaño‐Centellas 2020). Likewise, studies investigating how MSAGs respond to land use change typically find that networks become smaller, less cohesive, and less stable with increased fragmentation (Mokross et al. 2014; Goodale et al. 2015). Thus, we know that MSAGs vary across environmental gradients, yet how different environmental factors impact MSAG networks and why they vary is less clear.

**Fig. 1:**
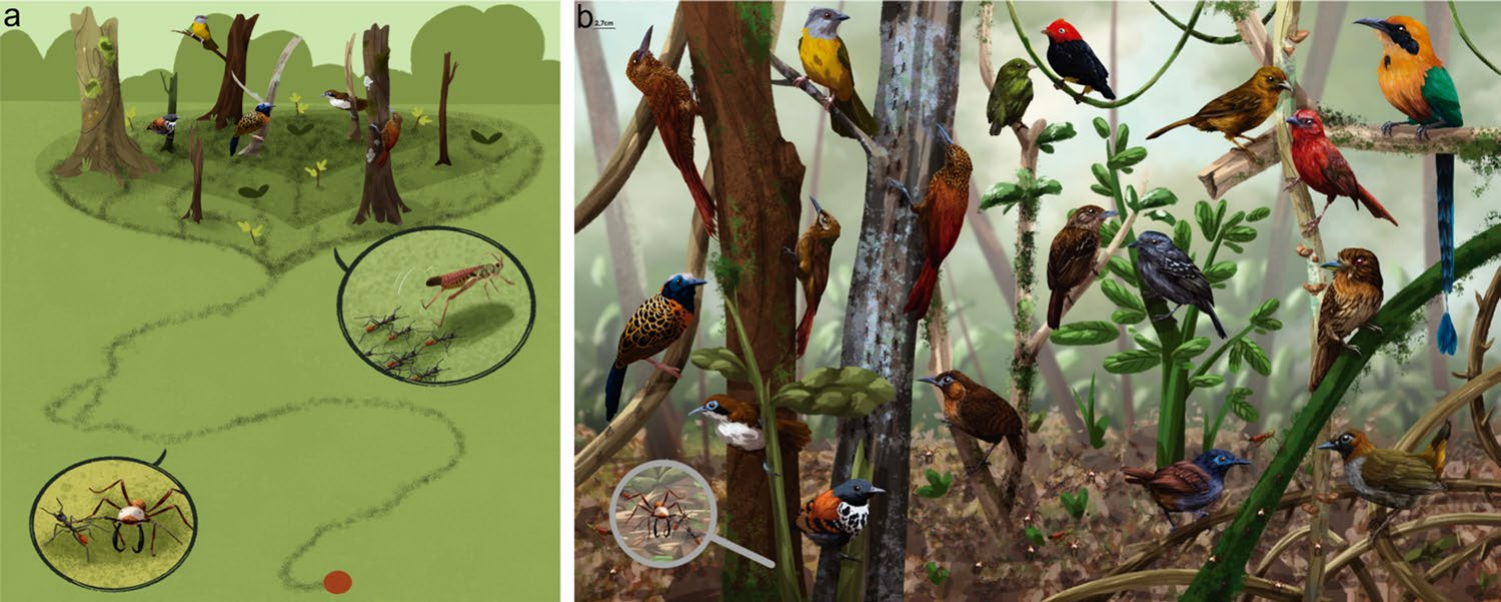
Illustrations of *E. burchellii* army ant swarms and the birds following them along the Panama Canal. **a** Drawing of army ants swarming and the birds following them. The close-up on the left side shows *E. burchellii* workers and soldiers and the close-up on the right side shows how the ants flush out arthropods. The top area represents the swarm with obligate and facultative bird species present. The red circle represents the ant nest (bivouac), and the black lines represent the swarming ants. **b** Drawing showing some of the diversity of bird species following *E. burchellii* swarms, with 109 bird species being observed at swarms along the Panama Canal alone (illustrations by J. A. Riascos-Ramírez)

We constructed ecological networks of one type of MSAG, army ant-following birds. These groups often include several individuals of multiple bird species that forage on the arthropods escaping from raiding army ants (Fig. 1). The size of the ant swarm and the presence of particular types of bird species (e.g., obligate species that specialize on foraging at swarms) play a role in the composition of birds found at swarms (Martinez et al. 2021; Gómez-Murillo 2022). In this study, we examined how environmental gradients influence the number of bird species (network size), the number (degree) and the frequency (weighted degree) of interspecific interactions, the tendency to form subgroups (clustering of species), and the skew of the frequency distribution of all species (skewness). Additionally, we examined the dissimilarity of networks across the gradients and evaluated whether dissimilarity was due primarily to the turnover of species or the rewiring of species (changes in how they interact). We examined two types of gradients—habitat suitability (i.e., fragmentation) and rainfall. These gradients were chosen because of the known impacts of rainfall and land use change on individual fitness, species distributions, population demography, and community structure (Lawrence and Vandecar 2015; Brawn et al. 2017; Betts et al. 2019; Boyle et al. 2020; Rutt et al. 2020). Understanding how multiple environmental gradients impact species interactions is critically important for predicting how species and biodiversity may change in the future. This is particularly true in the tropics, where over two-thirds of the world’s biodiversity exists, and at least 465 Neotropical bird species follow army ant swarms at least occasionally (Martinez et al. 2021).

## Material and methods

### Study species and sites

We conducted observations on birds attending swarms of *Eciton burchellii* (Hymenoptera: Formicidae: Ecitoninae); the army ants with the largest diurnal swarm raids (Gotwald 1996; Rettenmeyer et al. 2011). When swarming, *E. burchellii* move above-ground and follow a regular 5 week cycle consisting of 2 weeks of a nomadic phase and 3 weeks of a stationary phase, which allows the opportunity for birds to track and anticipate the ant’s behaviors (Swartz 2001). Ant nests, or bivouacs, are usually placed above ground, and certain bird species check on these bivouacs to ascertain whether the ants will be swarming that day (Swartz 2001; O’Donnell, et al. 2010).

We collected data for this study during 2020 (February and March) and 2021 (March to July) across the Isthmus of Panama (Online Resource Figure S1, Table S1). Weather is strongly seasonal in Panama, with a rainy season from April to December, and a marked dry season during the remainder of the year (Windsor 1992). In 2019 (April–August) and 2020 (February–March), we surveyed 15 sites across the Isthmus that varied in rainfall and amount of suitable habitat (see below). We searched for swarms at sites using standardized trail-walk surveys (Vidal-Riggs and Chaves-Campos 2008) and surveyed all sites every month across the study to look for swarms. Based on these surveys, we only found *E. burchellii* swarms present in seven of the sites (Online Resource Figure S1, Table S1). In 2021, we then focused our efforts on the seven sites where *E. burchellii* ant swarms were detected in previous years. In 2021, these sites were surveyed every 2 weeks. If a swarm was found during a survey, we would conduct observations that day and then track the swarm for multiple days if able (maximum of 3 days in a row). Individual sites had 6–27 days of swarm observations (except for one site that only had one swarm ever found, where we only had 1 day of data). *E. burchellii* swarms were not found at the lowest extreme of our habitat suitability gradient (4.8–30%). Nevertheless, the sites where *E. burchellii* swarms were present still varied in the amount of suitable habitat surrounding the site (from 31 to 99%, Online Resource Table S1) and mean annual rainfall (2103–2862 mm/year, see below on how rainfall data was derived).

### Habitat suitability and rainfall metrics

To measure fragmentation, we used a metric of habitat suitability that takes into account both habitat loss around a fragment and connectivity by including the amount of suitable habitat within a given distance around a site. This metric of habitat suitability was strongly correlated with fragment size (Spearman rank correlation = 0.9). To classify the percent of suitable habitat, we compared NDVI (normalized difference vegetation index) values between the fragments and area surrounding the fragment at the same time of year (Online Resource Figure S2). We assumed that the NDVI within a fragment where the ant swarm and the birds resided reflected suitable habitat and that large reductions in NDVI would reflect unsuitable habitat outside the fragment (e.g., tropical rainforest with high NDVI compared to a grassland with low NDVI). The amount of suitable habitat was defined as the proportion of NDVI values that matched a given fragment’s NDVI (within 10–90th percentiles) within a 200 m buffer area around the edge of the fragment. We chose the 10th and 90th percentiles to be conservative given that there is large variation in the NDVI values of what we would consider tropical forest. We selected a buffer of 200 m based on previous studies demonstrating that most understory birds cannot cross gaps larger than 100 m (Houtan et al. 2007; Stouffer, and Laurance 2004) and cannot cross open water more distant than 200 m (Moore et al. 2008). For example, a site embedded within a larger forest had a metric of 1, meaning that 100% of the habitat surrounding the site was suitable and it was highly connected. A highly isolated site had a metric closer to 0 (Online Resource Figure S2).

We used data from the rainfall monitoring program conducted by Panama Canal Authority Meteorology and Hydrology Stations. The Smithsonian Tropical Research Institute hosts these data (https://biogeodb.stri.si.edu/physical_monitoring/research/panamacanalauthority). Specifically, we calculated annual cumulative rainfall between 2009 and 2023 using the data collected by ~ 60 climatic stations (mean ± SD, 59.7 ± 3.1 stations year-1) distributed along the Panama Isthmus. We used these data, the geographic coordinates, and altitude above sea level of the stations, to fit a hierarchical Bayesian model to predict annual rainfall at the coordinates *XY* and altitude *K* across the Isthmus. See Online Resource Supplementary Methods for details. In summary, the model was well fitted and had a prediction accuracy of ~ 89% (88.6 ± 4.05%), so we were confident in using it to estimate average annual rainfall at each of our sampling sites.

### Locating ant swarms

To locate ant swarms, one to two observers (depending on the size of the site) searched each site systematically. Observers walked separately while carefully observing the ground for ant columns and listening for vocalizations of ant-following birds. In total, 92 *E. burchellii* swarms were observed, but 10 of these swarms had no birds attending, resulting in 82 swarms where bird observations were possible. Because of the nomadic behaviors of the ants, the small-scale heterogeneity in tropical forests, and likely shifts in many of the individual birds present as the swarm moved, the same ant swarm in a site was measured up to three times across three consecutive days (see below).

### Bird attendance at swarms

Once an ant swarm was located, observations were conducted by two observers at most swarms (89% of swarms). Single observers were used when the swarm was of relatively small size or with few to no birds attending the swarm. Observers positioned themselves 5–10 m from the edge of the front of the swarm at a location with an unobstructed view of the raid front. After approaching the swarm, we waited 5–10 min for birds to resume foraging activity before collecting data. Individual birds within 15 m from the edge of the swarm and actively catching insects being flushed by the ants were considered to be attending the ant swarm (Martínez et al. 2018). We conducted observations on the number of species of birds attending the swarm using a series of 5 min observation windows, with a minute of pause between them. We conducted an average of three 5 min periods per swarm (range: 2–6, 73% had 3 windows and 20% had 4) to ensure we had enough windows to generate a weighted network and that we were detecting all the species at the swarm in a single window.

### Network metrics

A single network is based on the group of birds following a single ant swarm on a given day. However, as the network is composed of multiple 5 min observation windows, those species that have not co-occurred in at least one time window will not be interacting in the final network (Figure S3). We built both binary and weighted networks. Binary networks were organized in the form of edge lists wherein links (interactions) are defined by the co-occurrence of two species at least once in an observation window (5-min window) in a given swarm (Figure S3). This resulted in binary networks which denote the presence or absence of an interaction (i.e., coexistence in at least one observation window) between species in a group. We also built weighted networks, where interaction frequencies of each pair of species represent how many time windows they coexisted in a group (Figure S3); these networks were only used for the metric of mean weighted degree (see below). We defined interspecific associations (i.e*.,* an interaction unity) based on group membership (i.e., gambit of the group), in which all bird species that coincided within a single 5-min observation window were considered to be associating reciprocally with one another (Whitehead and Dufault 1999; Rutt and Stouffer 2021). We consider networks based on co-occurrence to be a valid approach because it includes only species found in close proximity around a swarm (within 15 m of the swarm, Martinez et al. 2018). While the species that are found together at swarms may not always directly interact (such as directly fight with each other), they are foraging in close proximity and following the swarm together, similar to birds in mixed-species flocks. Further, we defined networks based on short-time windows, rather than collapsing all of this variation into one network per site given the dynamic nature of flocks. High variation in abundance and species present at swarms are found based on swarm traits, time of day, and the composition of bird species; thus, defining a network based on shorter time periods is a more realistic representation of the group of birds following a given swarm (Gómez-Murillo 2022). We acknowledge the possibility that we missed bird species in certain time windows, particularly the first time windows for a given swarm. Nevertheless, because we had two observers located in different parts of the swarm and upon arrival to the swarm, we would listen for 5–10 min for the bird species before starting the first time window, we believe that biases in detection are minimal (and similar across swarms). We analyzed networks using the R packages igraph (Csardi and Nepusz 2006) and moments (Komsta and Novomestky 2015).

For each network, we calculated five metrics, largely following Mokross et al. (2014) and Rutt and Stouffer (2021): (1) network size, (2) mean normalized degree, (3) mean weighted degree, (4) the global clustering coefficient (hereafter, clustering), and (5) skewness. Network size corresponds to the number of species attending the swarm (i.e., species richness). Mean normalized degree is the number of interspecific connections for a species, normalized by dividing by the number of available species (n-1) and then averaging across all species in the swarm. Thus, a higher mean normalized degree suggests each species associates with a larger proportion of other species. Mean weighted degree corresponds to the sum of the frequency of interspecific associations (edge weights) for each species, averaged across all species to obtain a single overall estimate for the network. Thus, a higher mean weighted degree suggests that the group of birds is more cohesive, with species both connected to many others and those connections are used more often. Clustering calculates the degree to which species tend to cluster together by measuring the tendency of species to form triangles (i.e., whether species that interact with the same species also interact with each other), thus, estimating how likely it is that two connected species are part of a highly connected group. Thus, higher clustering means birds tend to form tightly knit subgroups. Finally, skewness measures the skew of the frequency distribution of all species’ normalized degree values, or the extent to which a network has more species with few connections (positive skew) or many connections (negative skew). Thus, a more negative skew indicates almost all birds are highly connected, but a small number have few connections, while a skew closer to zero indicates a more even distribution of connections. Metrics were calculated using the R packages igraph (Csardi and Nepusz 2006) and moments (Komsta and Novomestky 2015).

Lastly, to evaluate differences in species associations across sites and test their association with environmental variation, we calculated network dissimilarity (β_WN_) between each pair of networks (3321 comparisons), and partitioned this value into its two additive components: dissimilarity associated with differences in species composition (β_ST_, i.e., turnover of species) and dissimilarity in interactions among shared species (β_OS_, i.e., rewiring of interactions) (Poisot et al. 2012). In brief, we used the function network_betadiversity of the R package betalink (Poisot et al. 2012) to calculate the interaction turnover between pairs of networks. Here, we calculated interaction dissimilarity using Whittaker’s equation (Whittaker 1960):

$$\beta WN = \frac{{\left( {a + b + c} \right)}}{{\left( {2a + b + c} \right)/2}} - 1,$$

wherein a is the count of shared interactions between networks b and c, b is the count of interactions unique to b, and c is the count of interactions unique to c. Each additive component was then divided by the total network dissimilarity (β_WN_) to find the proportion of the differences explained by either turnover of species or rewiring of interactions. These values were then used in our models to examine whether the variation in dissimilarity between swarms could be explained by the difference in mean annual rainfall or the difference in habitat suitability. Additionally, we calculated the average dissimilarity (β_WN_) and its two additive components (β_ST_ and β_os_) for each site (based on all dissimilarity values) to compare dissimilarity between each pair of sites, allowing one to visualize how different the sites are from each other.

### Analysis

All analyses were conducted in the R programming environment (R Core Team 2024, version 4.4.1).

*Network metrics* We used generalized linear mixed models (GLMMs) to analyze whether the variation in each network metric could be explained by the effects of mean annual rainfall or habitat suitability. We were unable to test for an interaction between the terms owing to *E. burchelli* swarms only being found at seven of our sites. Sites without swarms were not included in our dataset given we were interested in evaluating network typology, but are presented in Table S1. We used the following error distributions and link functions: Poisson with log-link function for network size, beta with logit-link function for mean normalized degree and clustering, and Gaussian for weighted degree and skewness. We tested the random effects of site, swarm id, month, observer, and swarm id nested within site and picked the final random effects structure based on which factor(s) explained any variance in the model. Only swarm_id explained variance in the model, and, therefore, was the random effect used. We used a standard AIC*c* multi-model selection approach to compare models with and without the quadratic effect of mean annual rainfall and chose the model with the lowest AICc. All fixed effects were scaled. We considered a fixed effect as significant if the p-value was ≤ 0.05. All models were implemented within the glmmTMB package (Brooks et al. 2017).

*Network dissimilarity* We used generalized additive mixed models (GAMMs) to analyze whether the variation in dissimilarity between swarms could be explained by the difference in mean annual rainfall or the difference in habitat suitability between the sites where swarms were located. We allowed both differences in rainfall and habitat suitability to be nonlinear. In GAMMs, the effective degrees of freedom (e.d.f.) determine the amount of nonlinearity; a value of 1 means the relationship between the fixed effect and the response variable is linear, and the higher the e.d.f, the more nonlinear the relationship is. In all models, we specified no more than three knots (owing to small sample sizes). We used “cs” as the basis function, which allows fixed effects to shrink to zero, meaning to make a flat line (*β* = 0), when there is no relationship between the fixed effect and the response variable. For overall dissimilarity, we used a beta error distribution with logit-link function, and for the turnover of species and species rewiring, we used Gaussian error distributions. All fixed effects were scaled, and in the case of species turnover and species rewiring, the response variables also needed to be scaled to improve model residuals. We considered the fixed effect to be important if the p-value was ≤ 0.05. GAMMs were implemented within the mgcv package (Wood et al. 2012). Pairwise comparisons are at the scale of single ant swarms, and therefore we included a random effect of the pair of sites in all models to account for nonindependence.

## Results

In total, 68 bird species were observed attending swarms across all swarms found (Table S3). More than half (58.8%) of the species attending *E. burchellii* swarms were encountered in only one to two of the seven sites. Additionally, 69.1% of the bird species attended only between 1 and 5 ant swarms of the 82 swarms included in the analyses, while only 26.5% of the species attended more than 5 (Table S3).

### Network characteristics

**Fig. 2:**
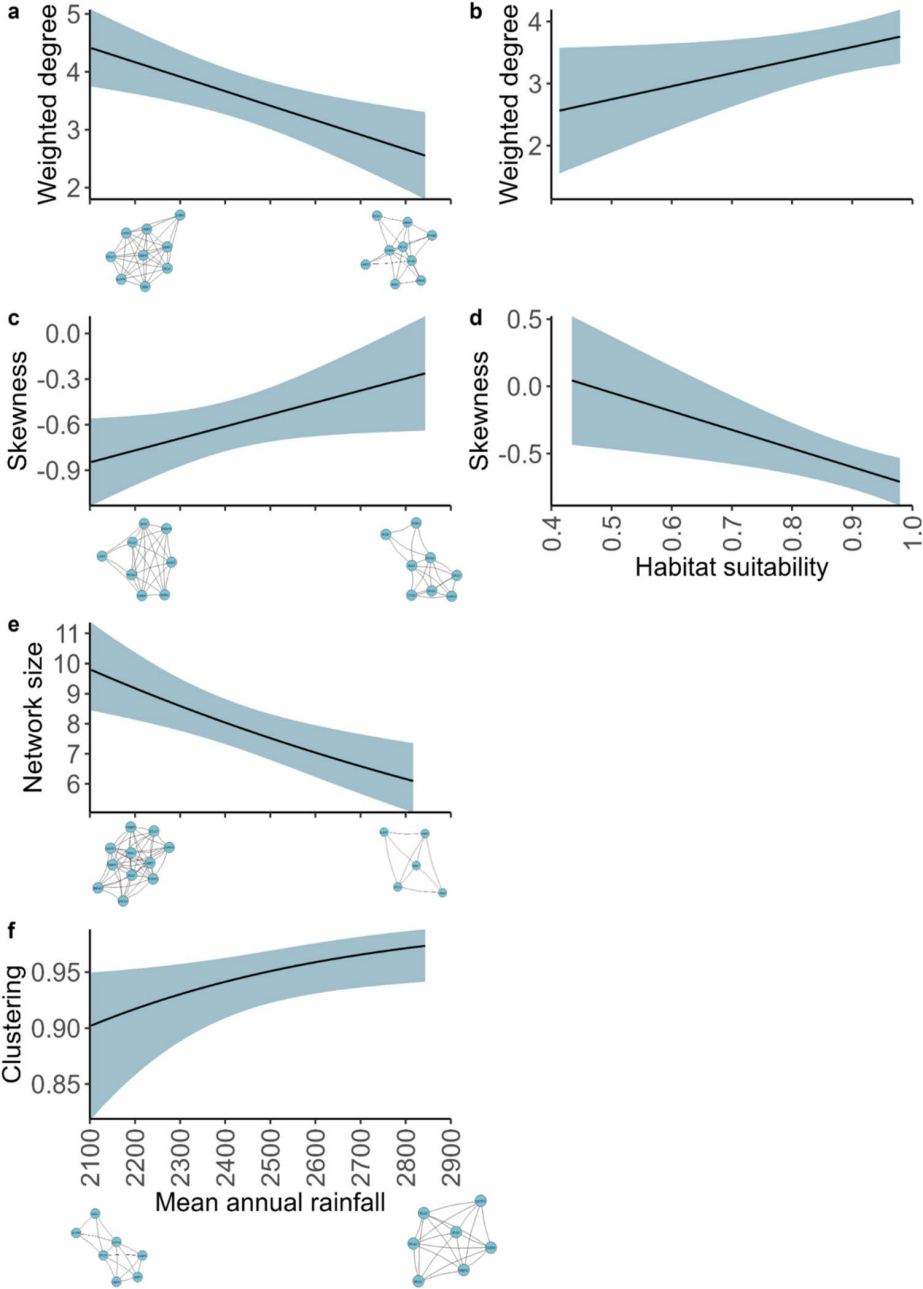
The influence of rainfall (**a**, **c**, **e**, **f**) and habitat suitability (**b**, **d**) on the network metrics of: **a**, **b** mean weighted degree, **c**, **d** skewness, **e** network size, and **f** clustering for birds attending *E. burchellii* swarms in Panama. Results are based on GLMM model output. 95% CIs are shown in the shaded region. Inset figures are example ant swarm networks with values similar to the extreme predicted values shown in the figures, controlling for network size (e.g., inset figures in (**a**) have a weighted degree of 4.89 (left) and 2.13 (right) and the same network size)

Four of the five network metrics were influenced by mean annual rainfall, and two were influenced by the amount of suitable habitat around the sites (Table 1). Ant-following birds had a higher mean weighted degree (stronger and more frequent associations) and greater network size (more species attending the swarms) in drier sites compared to wetter sites (Fig. 2a, e). Bird species formed more tightly knit subgroups (higher clustering) and skewness was less negative (the connections between species were more evenly distributed, with most species being highly connected) in wetter sites compared to drier sites (Fig. 2c, f). Nevertheless, clustering values were generally high and skewness values were always negative, indicating groups were generally clustered and species were well connected across all swarms found. The mean weighted degree was higher in sites with more suitable habitat (less fragmentation) and skewness was more negative in these same sites (Fig. 2b,d).

**Table 1:** Model results for (a) network size, (b) mean weighted degree, (c) clustering, (d) mean normalized degree, and (e) skewness

Response variable	Parameters	Beta estimate	Lower 95% CI	Upper 95% CI	*p*-value
(a) Network size	Intercept	2.065	1.971	2.160	0.000
	Annual rainfall	**− 0.176**	**− 0.279**	**− 0.073**	**0.001**
	Habitat suitability	0.093	− 0.006	0.192	0.139
(b) Mean weighted degree	Intercept	3.593	3.1972	3.9907	0.000
	Annual rainfall	**− 0.662**	**− 1.081**	**− 0.244**	**0.002**
	Habitat suitability	**0.441**	**0.027**	**0.855**	**0.037**
(c) Clustering	Intercept	2.830	2.358	3.301	0.000
	Annual rainfall	**0.493**	**0.060**	**0.926**	**0.026**
	Habitat suitability	− 0.018	− 0.445	0.409	0.934
(d) Mean normalized degree	Intercept	2. 765	2.271	3.258	0.000
	Annual rainfall	0.409	-0.054	0.871	0.083
	Habitat suitability	− 0.117	-0.565	0.331	0.609
(e) Skewness	Intercept	− 0.585	− 0.749	− 0.422	0.000
	Annual rainfall	**0.208**	**0.002**	**0.414**	**0.000**
	Habitat suitability	− **0.290**	− **0.490**	− **0.089**	**0.005**

Network metrics of birds attending *E. burchellii* ant swarms in Panama. 95% CIs that do not cross zero are in bold

### Network dissimilarity

**Fig. 3:**
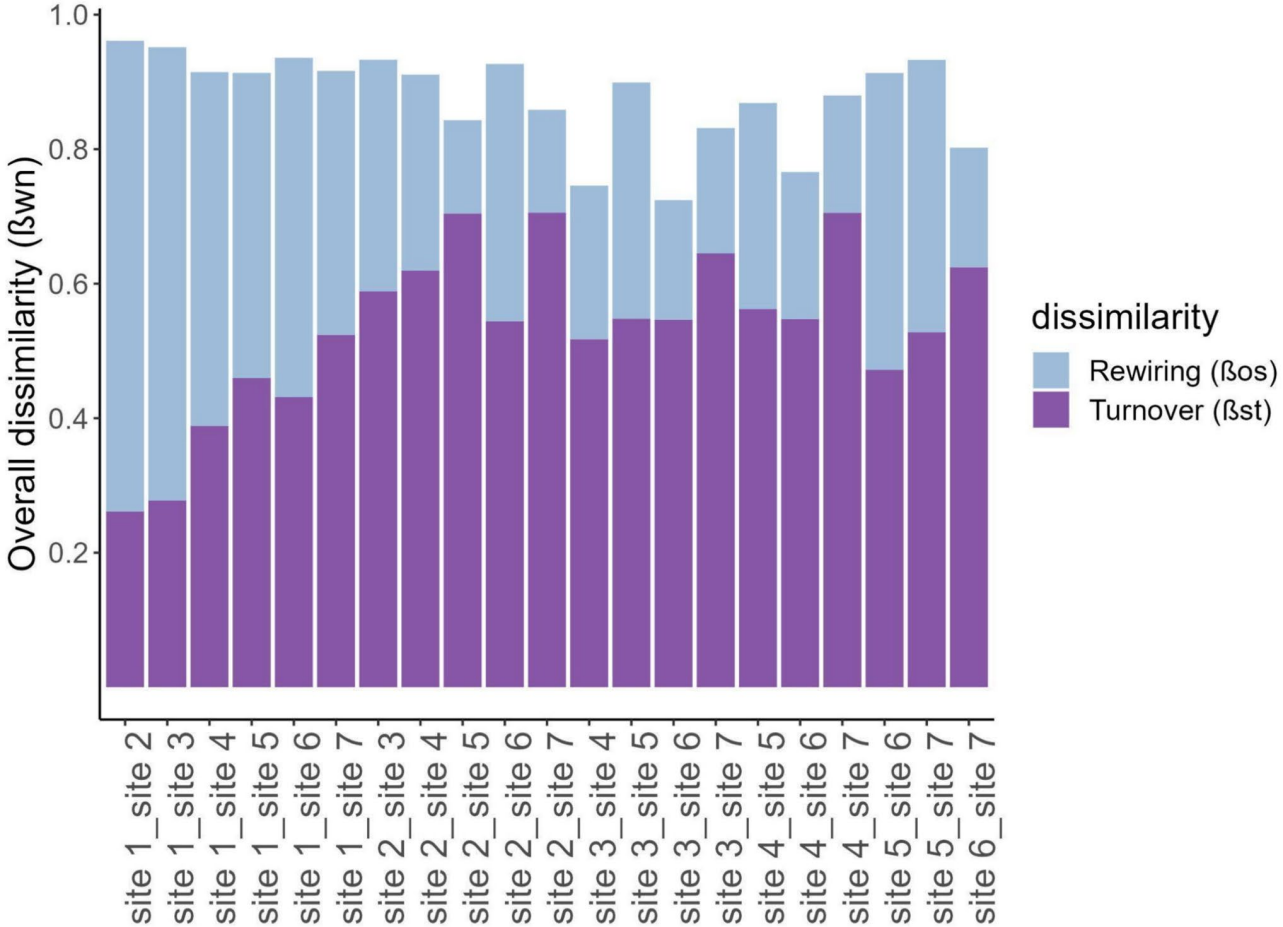
Average network dissimilarity (β_WN_) between pairs of sites, divided into its two additive components—dissimilarity associated with differences in species turnover (β_ST_, i.e., changes in the composition of species) in purple and dissimilarity in rewiring among shared species (β_OS_, i.e., changes in how species interact) in blue

We found a surprisingly high interaction dissimilarity among sites owing to both changes in species composition and rewiring. On average, only 11% of interactions were shared between sites (overall dissimilarity, β_WN_ = 0.89 ± 0.02, mean ± SE; *n* = 21 pairwise sites; Fig. 3), despite the most common bird species occurring at all sites. Approximately, 60% of the overall dissimilarity was associated with differences in species composition among sites (Species turnover, β_ST_ = 0.53 ± 0.03, mean ± SE; *n* = 21 pairwise sites; Fig. 3). In contrast, approximately 40% of the overall dissimilarity was because pairs of species that interacted in one site did not interact in another site where they co-occurred (species rewiring, β_OS_ = 0.34 ± 0.04, mean ± SE; *n* = 21 pairwise sites; Fig. 3).

**Fig. 4:**
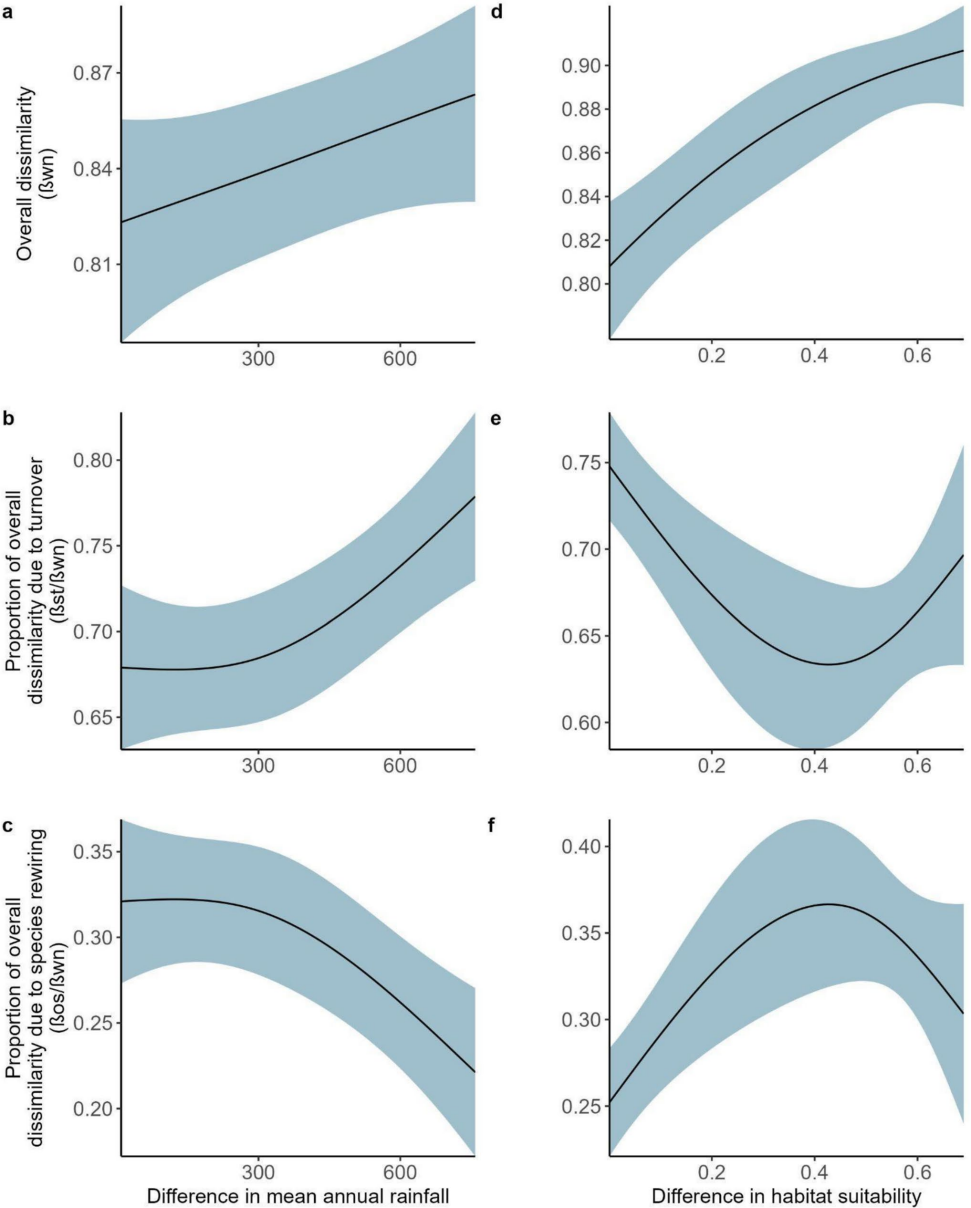
The influence of the difference in rainfall (**a**–**c**) and habitat suitability (**d**–**f**) between sites in **a**, **d** overall dissimilarity (β_WN_), **b**, **e** species turnover (β_ST_), and **c**, **f** species rewiring (β_OS_). Results are based on GAMM model output. 95% CIs are shown in the shaded region

Overall dissimilarity (β_WN_) was lower when the difference in rainfall between sites was lower and dissimilarity increased as the difference in rainfall between sites increased (Fig. 4a, Table 2). The overall dissimilarity across the rainfall gradient was due primarily to the turnover of species; sites with similar annual rainfall had lower species turnover (Fig. 4b, Table 2). Conversely, species rewiring was higher in areas with similar rainfall and lower in areas with differing rainfall (Fig. 4c, Table 2).

**Table 2:** Model results for (a) overall dissimilarity (β_WN_), (b) dissimilarity by species turnover (β_ST_), and (c) dissimilarity by rewiring (β_OS_) based on pairwise comparisons between swarms and a GAMM with pair of sites as a random effect

Response variable	Parameters	edf	*p*-value
(a) β_WN_	Difference in rainfall	**0.875**	**0.041**
	Difference in habitat suitability	**1.273**	**0.000**
(b) β_ST_	Difference in rainfall	**1.559**	**0.000**
	Difference in habitat suitability	**1.684**	**0.000**
(c) β_OS_	Difference in rainfall	**1.559**	**0.000**
	Difference in habitat suitability	**1.684**	**0.000**

Significant values are in bold

The amount of suitable habitat also influenced overall dissimilarity; smaller differences in habitat suitability corresponded with lower overall dissimilarity, and dissimilarity generally increased as the differences in habitat suitability increased (Fig. 4d, Table 2). Species turnover was lowest at sites with intermediate differences in habitat suitability (Fig. 4e, Table 2). These sites are where the rewiring of species was highest, while lower dissimilarity due to rewiring was found at the extremes of the habitat suitability gradient (Fig. 4f, Table 2).

## Discussion

By studying the mixed-species group of birds following *E.burchellii* swarms along environmental gradients in Panama, we found the following key results. First, network structure of ant-following birds changed along the rainfall gradient, with networks differing in size, complexity, and cohesion across the gradient. Second, habitat suitability also influenced network structure in the sites where ant swarms were found, and in areas with lower habitat suitability, there was the total dissolution of these groups. Third, the interaction dissimilarity between sites was exceptionally high in this system, owing to both turnover in species and rewiring. This indicates changes in species composition across the environmental gradients and a large pool of bird species that take advantage of the presence of ant swarms. Fourth, within sites, bird species that follow army ant swarms are well connected to each other, forming cohesive groups that interact repeatedly. This indicates the importance of ant swarms to a diversity of species. Overall, our results indicate the importance of rainfall and habitat suitability in structuring mixed-species groups and that ant-following birds form highly cohesive groups, making them potentially less resilient to changes in the environment and the loss of particular species.

### Network structure across gradients

Our analyses revealed that rainfall strongly influenced network structure in groups of ant-following birds in lowland forests of Central Panama. In drier sites, more species of birds joined ant swarms and the groups were more cohesive; the strength of interactions between species was greater, there were more well-connected species overall, and species formed tighter subgroups compared to wetter forests.

Variation in network structure along the rainfall gradient likely reflects food availability, with greater benefits of attending swarms, and thus larger and more cohesive networks in forests with lower food abundance and insect activity. In drier and more seasonal tropical forests, arthropod abundance tends to be lower (Prather et al. 2020) due to higher desiccation risk (Coley and Barone 1996; Givnish 1999; Connahs et al. 2011) and lower plant productivity (Coley and Barone 1996; Richards and Coley 2007). Conversely, insect abundance and activity tend to be higher in wetter forests due to greater soil and litter moisture and higher primary production (Levings and Windsor 1984; Kaspari and Weiser 2006; Brenes-Arguedas et al. 2009; Prather et al. 2020). One of the primary benefits of birds joining MSAG is increased food consumption and foraging efficiency. Thus, the benefits of joining these groups is likely to be higher under lower quality foraging conditions (Goodale et al. 2017a, b; Nauta et al. 2021; Rutt and Stouffer 2021; Richardson et al. 2022). Avian species richness is greater on the wetter side of the Isthmus (Rompré et al. 2007). Therefore, smaller network size in wetter sites is not due to lower avian diversity, but rather likely reflects better overall resource conditions, and less reliance on swarms, on the wetter side of the gradient.

Often when network size increases, groups are less cohesive and the strength of interactions becomes weaker (Fontaine 2013; Welti and Joern 2015). For example, Rutt and Stouffer (2021) showed that during the dry season, mixed-species bird flocks had more species, but were less cohesive and species were less well connected (lower mean weighted degree, positive skew, lower clustering). We did not find this pattern here, however. We found that even though groups were larger in the drier forests, species were more connected and interacted with each other more frequently in these forests compared to wetter forests. This may arise because more bird species benefit from attending swarms in drier forests, and thus the species that benefit from attendance tend to recruit to and stay at these swarms (Richardson et al. 2022). In contrast, in wetter forests, where the benefits of attendance may be lower, there may be more occasional visitors to swarms and less reliance on army ant swarms for food, resulting in less cohesion and a lower frequency of interactions among species. Nevertheless, further work needs to be done to evaluate differences in food availability across the rainfall gradient, and how the costs and benefits of attending army ant swarms varies with rainfall.

Habitat suitability altered network structure; sites with more suitable habitat had stronger and more frequent associations that were more well connected compared to sites with less suitable habitat. We hypothesize that these patterns arise because the abundance of species important for recruitment to swarms (e.g., the obligate species) are greater in sites with more suitable habitat (Mokross et al. 2014; Martinez et al. 2018; Martinez et al. 2021), resulting in these groups being more cohesive when particular species are present. For example, we found that the average abundance of obligates at a single swarm was 5.7 at sites with more suitable habitat (> 90% suitable habitat, average abundance ranging from 3 to 7.5, depending upon the site), while the average abundance was 0.05 at sites with less suitable habitat (< 50% suitable habitat, range: 0–0.08, depending upon the site) (Gómez-Murillo, unpubl. data).

The impact of habitat suitability on network structure was weaker than the impact of rainfall. This could indicate that the networks of ant-following birds are somehow resilient to habitat changes. However, the fact that we did not find any *Eciton burchellii* army ants in sites with lower habitat suitability (< 31%, Table S2) indicates that this community may collapse when there is a low amount of suitable habitat (Mokross et al. 2014). However, we did detect *Labidus praedator* ant swarms, the other diurnal army ant species in Panama, at sites with less suitable habitat (Gómez-Murillo 2022). While birds will attend *L. praedator* swarms, fewer individuals and species attend these swarms (on average, 4 species and 6 individuals), obligate species are rarely in attendance (on average, 0.5 individuals), and these swarms are an ephemeral and less dependable resource compared to *E. burchellii* (Willson 2004; Gómez-Murillo 2022). Thus, *L. praedator* swarms do not maintain the same community of birds as *E. burchellii* swarms. Overall, we suggest that the weaker effect of habitat suitability on network structure of birds following *E. burchellii swarms* is because low habitat suitability (and thus smaller fragment sizes) resulted in the total dissolution of these groups. Declines of *E. burchellii* army ant populations due to forest fragmentation have been documented in several studies (Roberts et al. 2000; Meisel 2006; Berhoff et al. 2008; Peters and Okalo 2009; Willson et al. 2011). Large home ranges and high mobility may make it difficult for army ants to persist in small fragments, especially where matrix conditions are hostile (Stouffer and Bierregaard 1995; Laurance et al. 2002). Thus, network dissolution of *E. burchellii* swarms appears to occur in smaller and poorly connected fragments.

### Dissimilarity across gradients

The high dissimilarity observed in ant swarm networks across environmental gradients in Panama was similar to those reported in mutualistic (pollination and seed dispersal) networks (Carstensen et al. 2014; Vizentin-Bugoni et al. 2019), and was similar or greater than that found in mixed-species bird flocks across elevational gradients (Montano-Centellas et al. 2020). This high dissimilarity across ant swarm networks suggests that there are large changes in species composition across our gradients, a large suite of bird species join army ant swarms to forage, and species vary in whether they interact within swarms (i.e., rewiring). Species composition of mixed-species groups changes with the composition of the overall bird community (Hutto 1994; Péron and Crochet 2009; Goodale et al. 2010), and in tropical locations, species ranges tend to be narrow (Jankowski et al. 2010). Correspondingly, 58.8% of the species we observed attending *E. burchellii* swarms were encountered in only one to two of the seven sites (Table S3). While we did not evaluate whether changes in species composition were due to the replacement of species or nestedness, we do know that network size did not change with habitat suitability and there are changes in which species are present at different sites across the rainfall gradient (Rompré et al. 2007). The high turnover of species across the short 65-km rainfall gradient supports work indicating high turnover in tropical bird communities across short distances (Jankowski et al. 2009; Gomez et al. 2020) and that at army ant swarms, most of the species associations are unique. Further, we have observed over 100 species attending ant swarms along our gradients, highlighting that a large number of the bird community will attend swarms to find food. Rewiring also influenced dissimilarity across sites, although to a lesser degree than species turnover. Rewiring may occur because most species attending swarms are non-obligates and thus whether they attend may be context-dependent. For example, previous work found that the number of obligate individuals at a swarm and swarm size influence the species found at swarms (Gomez-Murrillo 2022).

Overall dissimilarity and species turnover were greater when differences in rainfall were greater, indicating the importance of rainfall on species distributions. The magnitude of rainfall effects on species distributions often equals or exceeds that of temperature effects in both temperate and tropical environments (Bonebrake and Mastrandrea 2010; Gomez et al. 2018). Distributions of terrestrial animals are likely shaped by combinations of direct, physiological responses to rainfall and indirect responses, mediated by species interactions (Barton et al. 2019; Boyle et al. 2020). For example, interactions between plant-pollinator communities are mediated by rainfall: as bird pollination tends to increase with an increase in rainfall, insect pollination tends to decrease (González et al. 2009). Additional bottom-up processes, such as food availability, may connect rainfall to survival or reproduction at extremes of the rainfall gradient (Boyle et al. 2020), altering the initial species pool and the abundance of ant-follower birds at the edges of the gradient. Our results suggest that rainfall indirectly affects species interactions by impacting species distributions.

Dissimilarity between networks was also influenced by the amount of suitable habitat, with overall dissimilarity increasing as the differences in habitat suitability between sites increased. Similar to rainfall, differences in the networks across sites were primarily driven by species turnover in sites differing in habitat suitability (rather than by rewiring). The effect of fragmentation and land use change in bird communities is well documented and ranges from influencing behavior, population demography, community structure, to ecosystem function (Laurance et al. 2004; Cuervo and Restrepo 2007; Sekercioglu and Sodhi 2007; Ruiz‐Guerra et al. 2012; Rutt et al. 2020; Stouffer 2020). In particular, insectivorous tropical birds are known to be highly sensitive to changes in fragment size (Powell et al. 2013; Stouffer 2020). Here, we would argue that habitat suitability influenced which species of birds were present at these sites, resulting in high dissimilarity across sites and the same species only being found in one to two sites across our gradients (Table S3).

### Conservation implications

Despite only a handful of highly specialized bird species (obligates) at swarms and many of the bird species only following ant swarms on occasion, we found higher clustering and more well-connected (skewness was more negative) networks compared to many studies on mixed-species flocks (Mokross et al. 2014; Goodale et al 2020; Montano-Centellas et al. 2020; Rutt and Stouffer 2021). This suggests that the group of birds that follow army ant swarms may be less resilient to the loss of particular species or to environmental changes than mixed-species flocks and other ecological networks. Previous work found the important role of obligate species in advertising the presence of ant swarms to other species (Martinez et al. 2018) and that too few obligate individuals are associated with a reduction in the number of species and individuals following swarms (Gómez-Murillo 2022). Thus, the loss of these particularly important species may result in a breakdown in these groups, with potentially cascading impacts on non-obligate species.

More well-connected groups suggest that despite primarily non-obligate species attending ant swarms, ant swarms are an important, shared food resource for bird species, drawing together a diverse and tightly connected group of birds. While in the lowland forests along the Panama Canal, there are only 3 obligate species, we have detected 107 other bird species attending ant swarms (here and De Aquino, unpubl. data). Thus, there is an incredible diversity of bird species that can take advantage of this slow moving food resource, including birds that differ greatly in their foraging behaviors (foraging from the ground to 15 m up, varying in perch types, foraging maneuvers, and prey consumption) and body size (6–400 g). This food resource may be particularly important to a large proportion of the avian insectivore community, particularly when food is scarce. While more work is needed to understand the role of the environment in these groups and how the loss of the ants or obligate species impacts the non-obligate community, our results highlight the important role of *E. burchellii* swarms to the avian community.

Although interaction networks change along environmental gradients (Devoto et al. 2005; Mokross et al. 2014; Welti and Joern 2015; Tylianakis and Morris 2016; Pellissier et al. 2018; Montaño‐Centellas 2020), most networks have been studied as static entities at single sites. Nevertheless, multi-site studies are critically important for understanding the processes underlying network structure and for evaluating the generalizability of network patterns (Dáttilo et al. 2019; Vizentin-Bugoni et al. 2019). Mixed-species animal groups have been proposed as ‘conservation targets’, as they are easily monitored and susceptible to anthropogenic disturbance (Zou et al. 2018). Interspecific interactions in communities are an essential component of ecosystem function and have important implications for the ecological and evolutionary dynamics of species (Mokross et al. 2014). The results presented here advance our understanding of how non-trophic interspecific interactions and subsequent community structure change along environmental gradients.

## Supplementary Information

Below is the link to the electronic supplementary material.

Supplementary file1 (DOCX 5404 KB)

## Data Availability

The datasets used in this article are available through Harvard dataverse repository (10.7910/DVN/MPHKZN)

## Code availability

Code used to analyze the data in this article are available through Harvard dataverse repository (10.7910/DVN/MPHKZN)
