# Supplementary material for: Rainfall alters network structure, while fragmentation results in the breakdown of a mixed-species group of birds
Source: Oecologia. 2026 Feb 7;208(2):34. doi: 10.1007/s00442-026-05869-7 (PMC12882967; doi:10.1007/s00442-026-05869-7)
Supplement: Supplementary file 1 — Supplementary file1 (DOCX 5404 KB) [file 442_2026_5869_MOESM1_ESM.docx]

**Electronic Supplementary Material**

**Table S1.** Detailed information for each of the 15 field sites sampled along the Isthmus of Panama during 2020 and 2021. Included is the presence of obligate species at a given site (bicolored antbird, *Gymnopithys bicolor*, or ocellated antbird, *Phaenostictus mcleannani*). We observed *E. burchellii* swarms at only seven of the sites.


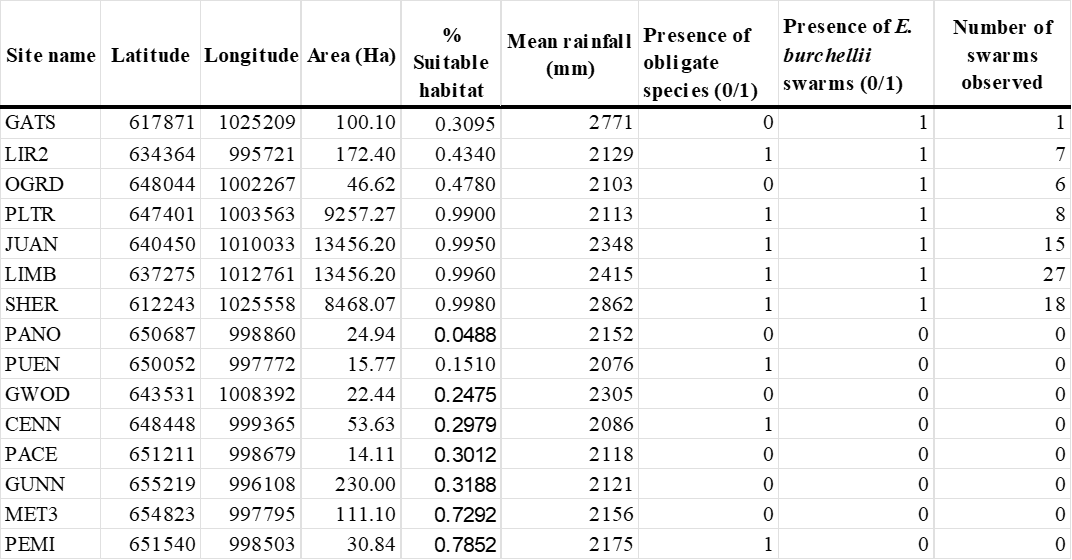


**Table S2.** Summary of network metrics.

| **Variable** | **Minimum** | **Median** | **Mean** | **Maximum** |
| --- | --- | --- | --- | --- |
| Network size | 2.0000 | 8.0000 | 8.1341 | 14.0000 |
| Mean normalized degree | 0.5400 | 0.9000 | 0.8851 | 1.0000 |
| Mean weighted degree | 0.9100 | 3.4750 | 3.6948 | 7.6400 |
| Skewness | -2.4845 | -0.7071 | -0.6071 | 1.0206 |
| Global clustering coefficient | 0.5900 | 0.9100 | 0.8986 | 1.0000 |

**Table S3.** Species observed attending *E. burchellii* ant swarms in Panama (Willis and Oniki 1978, Swartz 2001, Martínez et al. 2018).

| **# of species** | **Scientific name** | **English name** | **# of swarms** | **# Sites** |
| --- | --- | --- | --- | --- |
| 1 | *Accipiter_superciliosus* | Tiny Hawk | 1 | 1 |
| 2 | *Arremon_aurantiirostris* | Orange-billed Sparrow | 2 | 1 |
| 3 | *Attila_Spadiceus* | Bright-rumped Attila | 3 | 2 |
| 4 | *Baryphthengus_martii* | Rufous Motmot | 7 | 4 |
| 5 | *Cacicus_uropygialis* | Scarlet-rumped Cacique | 2 | 2 |
| 6 | *Campephilus_melanoleucos* | Crimson-crested Woodpecker | 1 | 1 |
| 7 | *Cantorchilus_leucotis* | Buff-breasted Wren | 1 | 1 |
| 8 | *Cantorchilus_nigricapillus* | Bay Wren | 5 | 1 |
| 9 | *Catharus_ustulatus* | Swainson Thrush | 1 | 1 |
| 10 | *Celeus_loricatus* | Cinnamon Woodpecker | 4 | 2 |
| 11 | *Ceratopipra_mentalis* | Red-capped Manakin | 22 | 6 |
| 12 | *Cercomacroides_tyrannina* | Dusky Antbird | 4 | 3 |
| 14 | *Chiroxiphia_linearis* | Long-tailed manakin | 1 | 1 |
| 15 | *Crotophaga_major* | Greater Ani | 1 | 1 |
| 16 | *Cyanoloxia_cyanoides* | Blue-black Grosbeak | 1 | 1 |
| 17 | *Cymbilaimus_lineatus* | Fasciated Antshrike | 4 | 2 |
| 18 | *Cyphorhinus_phaeocephalus* | Song Wren | 21 | 5 |
| 19 | *Dendrocincla_fuliginosa* | Plain-brown Woodcreeper | 63 | 7 |
| 20 | *Dendrocincla_homochroa* | Ruddy Woodcreeper | 2 | 1 |
| 21 | *Dendrocolaptes_sanctithomae* | Northern barred-woodcreeper | 41 | 5 |
| 22 | *Dysithamnus_puncticeps* | Spot-crowned antvireo | 1 | 1 |
| 23 | *Electron_platyrhynchum* | Broad-billed Motmot | 6 | 3 |
| 24 | *Empidonax_virescens* | Acadian Flycatcher | 3 | 3 |
| 25 | *Epinecrophylla_fulviventris* | Checker-throated Stipplethroat | 3 | 3 |
| 26 | *Eucometis_penicillata* | Grey-headed Tanager | 49 | 7 |
| 27 | *Euphonia_laniirostris* | Thick-billed Euphonia | 1 | 1 |
| 28 | *Formicarius_analis* | Black-faced Antthrush | 14 | 4 |
| 29 | *Gymnopithys_bicolor* | Bicolored Antbird | 66 | 5 |
| 30 | *Habia_fuscicauda* | Red-throated Ant-tanager | 12 | 5 |
| 31 | *Henicorhina_leucosticta* | White-breasted Wood-Wren | 3 | 3 |
| 32 | *Hylophylax_naevioides* | Spotted Antbird | 43 | 5 |
| 33 | *Lepidothrix_coronata* | Blue-crowned Manakin | 7 | 4 |
| 34 | *Malacoptila_panamensis* | White-whiskered Puffbird | 5 | 3 |
| 35 | *Manacus_vitellinus* | Golden-collared Manakin | 6 | 5 |
| 36 | *Microbates_cinereiventris* | Tawny-faced Gnatwren | 1 | 1 |
| 37 | *Microrhopias_quixensis* | Dot-winged Antwren | 6 | 3 |
| 38 | *Mionectes_oleagineus* | Ochre-bellied Flycatcher | 4 | 3 |
| 39 | *Momotus_subrufescens* | Whooping Motmot | 4 | 1 |
| 40 | *Myiornis_atricapillus* | Black-capped Pygymy-Tyrant | 2 | 1 |
| 41 | *Myrmotherula_axillaris* | White-flanked Antwren | 3 | 3 |
| 42 | *Neomorphus_geoffroyi* | Rufous-vented Ground-Cuckoo | 1 | 1 |
| 43 | *Oncostoma_olivaceum* | Southern Bentbill | 7 | 3 |
| 44 | *Onychorhynchus_coronatus* | Royal Flycatcher | 1 | 1 |
| 45 | *Pachyramphus_polychopterus* | White-winged Becard | 1 | 1 |
| 46 | *Phaenostictus_mcleannani* | Ocellated antbird | 51 | 3 |
| 47 | *Phaethornis_striigularis* | Stripe-throated Hermit | 1 | 1 |
| 48 | *Phaethornis_superciliosus* | Long-tailed hermit | 2 | 2 |
| 49 | *Pheugopedius_fasciatoventris* | Black-bellied Wren | 3 | 1 |
| 50 | *Pheugopedius_rutilus* | Rufous-breasted Wren | 1 | 1 |
| 51 | *Poliocrania_exsul* | Chestnut-backed Antbird | 11 | 4 |
| 52 | *Ramphastos_ambiguus* | Yellow-throated Toucan | 3 | 3 |
| 53 | *Ramphastos_sulfuratus* | Keel-billed toucan | 3 | 2 |
| 54 | *Ramphocaenus_melanurus* | Long-billed Gnatwren | 1 | 1 |
| 55 | *Rhynchocyclus_olivaceus* | Olivaceus Flatbill | 2 | 2 |
| 56 | *Sclerurus_guatemalensis* | Scaly-throated Leaftosser | 1 | 1 |
| 57 | *Terenotriccus_erythrurus* | Ruddy-tailed Flycatcher | 1 | 1 |
| 58 | *Thamnophilus_atrinucha* | Black-crowned Antshrike | 38 | 6 |
| 59 | *Thryophilus_rufalbus* | Rufous-and-white Wren | 2 | 1 |
| 60 | *Tinamus_major* | Great Tinamu | 2 | 1 |
| 61 | *Trogon_chionurus* | White-tailed Trogon | 1 | 1 |
| 62 | *Trogon_massena* | Slaty-tailed Trogon | 3 | 2 |
| 63 | *Trogon_melanurus* | Black-tailed Trogon | 1 | 1 |
| 64 | *Trogon_rufus* | Black-throated Trogon | 2 | 2 |
| 65 | *Turdus_grayi* | Clay-colored Thrush | 1 | 1 |
| 66 | *Xenops_minutus* | Plain Xenops | 1 | 1 |
| 67 | *Xiphorhynchus_lachrymosus* | Black-striped Woodcreeper | 6 | 2 |
| 68 | *Xiphorynchus_susurrans* | Cocoa Woodcreeper | 57 | 7 |


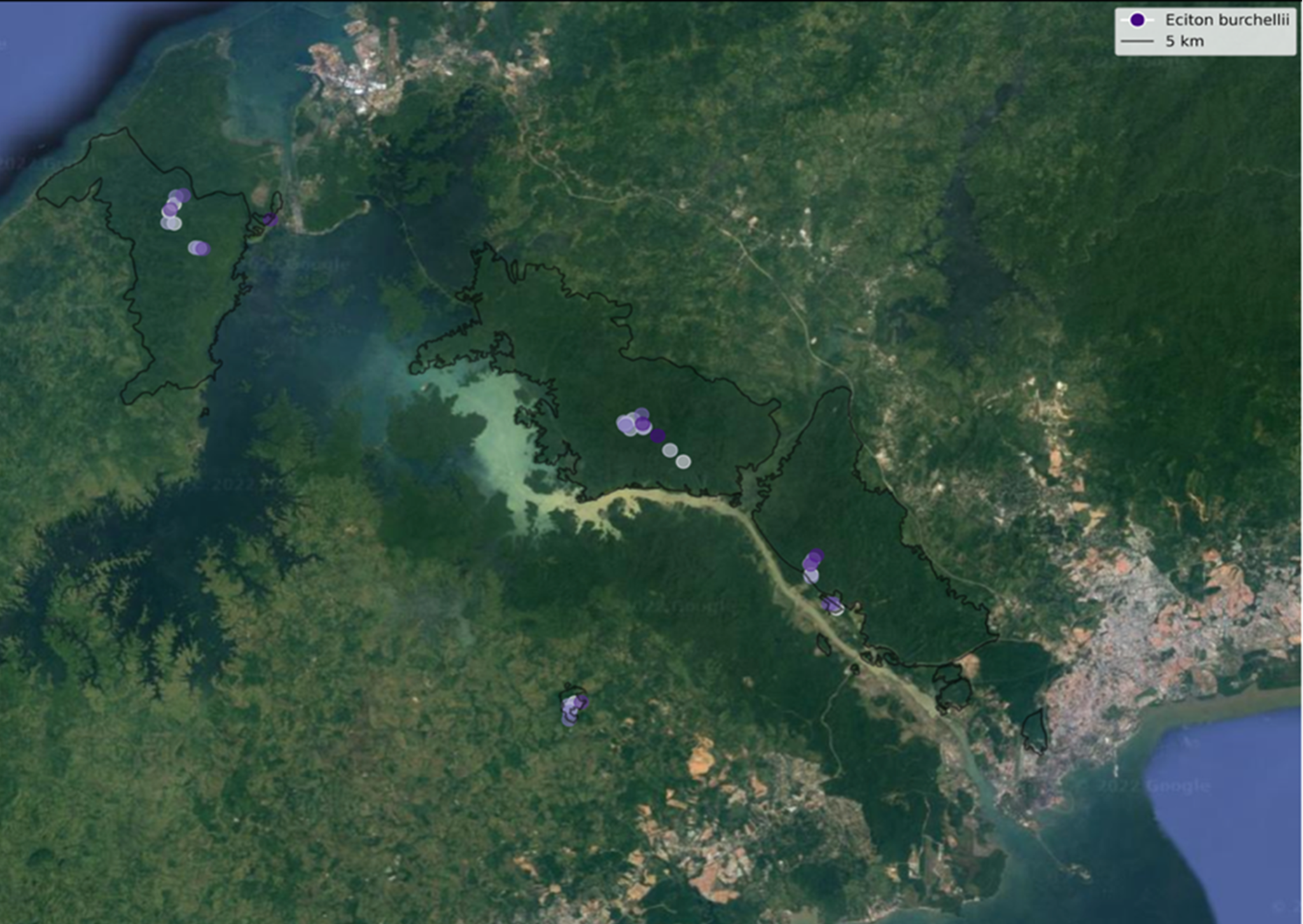


**Figure S1.** Location of the sites along the Isthmus of Panama. Perimeters of the sites are plotted in black at actual size along the Panama Canal. Purple dots represent each swarm of *E. burchellii* found (created by David M. DeFilippis).



**Figure S2.** Two study sites exemplifying the classification metric of habitat suitability surrounding each site. Left, a highly isolated site with only 4.8% habitat suitability around the 200m buffer band. Right, site embedded within a larger forest with 100% habitat suitability around the 200m buffer band.


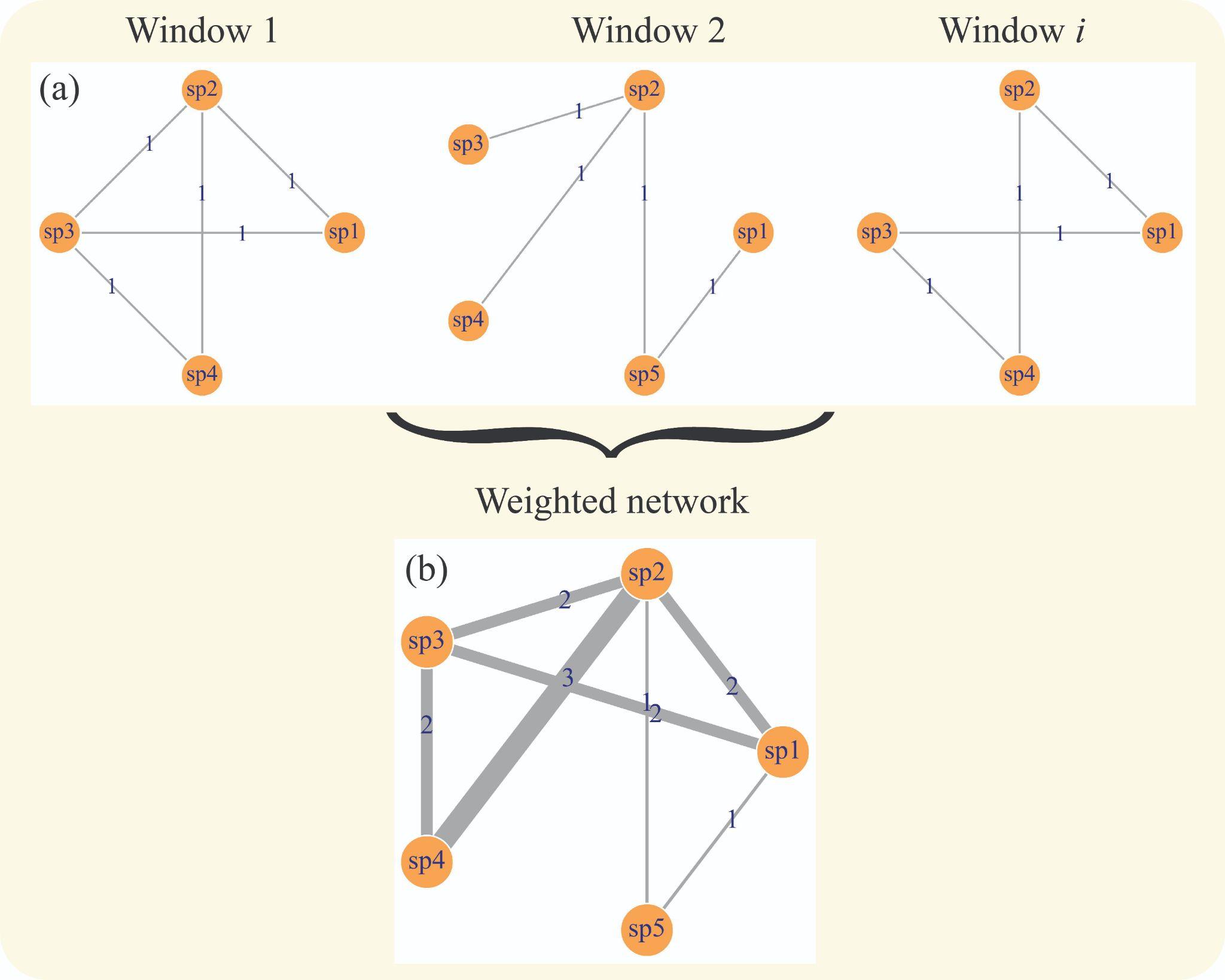


**Figure S3.** Process to construct weighted networks of ant-follower birds. **a)** Co-occurrence of species within group **j** in a given temporal window **k** denotes interactions among species. **b)** Pooling together co-occurrence data from all temporal windows of group **j** was employed to construct weighted interaction networks. Edge thickness and numbers indicate the frequency of interactions between pairs of species. Binary networks were constructed in the same way, based on the co-occurrence of species in at least one temporal window.

**Supplementary Methods.**

# **1. Introduction**

This document is divided into two sections: (i) an explanatory text of the procedure for fitting the model estimating annual rainfall at Panama’s Isthmus for the period 2009-2023; (ii) the R code needed to fit the model. Section ii is organized into sub-sections explaning the procedures for extracting observed rainfall, data wrangling processes, model fitting and diagnostic, testing prediction performance, and actual prediction of annual rainfall.

# **2. Section i**

*Annual rainfall data* $-$ We used historical data from the rainfall monitoring program conducted by Panama Canal Authority Meteorology and Hydrology Stations. The Smithsonian Tropical Research Institute hosts this data (<https://biogeodb.stri.si.edu/physical_monitoring/research/panamacanalauthority>). First, we used all the climatic stations distributed along the Panama channel (N = 74) and calculated the number of stations with annual data available between 1880 and 2023. Thus, we identified the time series interval with the highest number of stations per year and with raw data available. This process led us to the interval between 2009 and 2023, which implied 60 climatic stations per year on average (mean $\pm$ SD, 59.7 $\pm$ 3.1 stations $year^{-1}$). Finally, we used the raw data and calculated the annual rainfall per station.

*Estimating annual rainfall* $-$ Our goal was to estimate annual rainfall during the year i, coordinates XY, and altitude K at the Panama isthmus. To do so, we fitted the following Bayesian hierarchical model:

$${Annual rainfall}_{coordinates XY} \sim Student t(\nu= 2, \mu_{i}, \sigma)$$

$$\mu_{i}= \alpha_{year i} + \tau_{station i} + \beta_{1} \times{UTM N}_{i} +\beta_{2} \times{UTM W}_{i} + \beta_{3} \times{altitude}_{i}$$

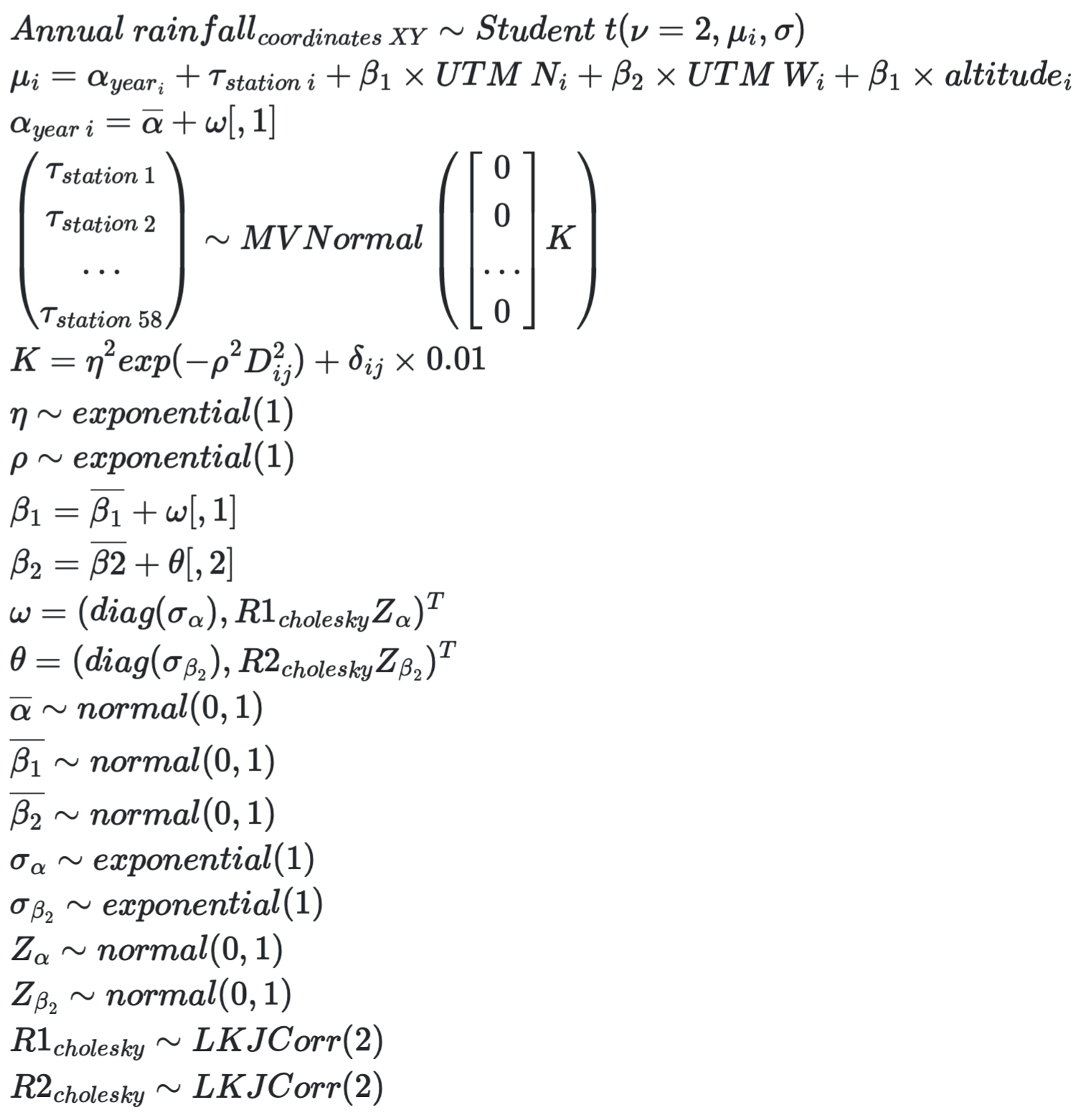


We fitted the model using z-scores of continuous response and predictor variables and used a Student-t probability distribution as the likelihood function to model annual rainfall. We used an MVnormal distribution to conduct partial pooling across 𝛼 and β parameters to account for potential covariation among slopes and intercepts across the years. Moreover, we used Gaussian processes to conduct partial pooling across stations (i.e., $\tau$ parameter) and account for their spatial correlation. To do so, we employed a quadratic kernel to construct the covariance matrix of an MVNormal distribution to estimate correlated parameters (McElreath 2020), and used as input a matrix of spatial distance among climatic stations. We used skeptical priors of strong effects, parameterized to encompass values within the possible range of annual rainfall. To set up the Hamiltonian MCMC algorithm, we defined three chains, 3000 sampling and 500 warming iterations, and a thinning rate of 3. Finally, we conducted sampling diagnostics of the model through visual assessment of chain convergence, Rhat < 1.1 and ess > 1000. We also assessed the quality of the models fit through posterior predictive checks.

Once we verified that the model was correctly fitted, we conducted random partitions of the data in training (80%) and testing (20%). Then we fitted the model each time to estimate the effect of the partition on the prediction performance of the model. First, we used UTM coordinates, altitude, and years of the training data to estimate annual rainfall. Then, we identified the four nearest stations to each specific location and marginalized their parameters to increase the prediction accuracy. Finally, we calculated the percentage of prediction error by analyzing the differences between observed and predicted annual rainfall. The model predicted annual rainfall with an 88% of accuracy (88.6 $\pm$ 4.05 percentage of accuracy), which implies that our model over or underestimated annual rainfall by 103 mm (103.4 $\pm$ 80.2 mm). Therefore, after testing its performance, we use this model to estimate annual rainfall at our sampling sites. We used R 4.4.0 (R Core Team, 2024) and package cmdstanr (Gabry et al. 2024) as the interface to run the Hamiltonian MCMC algorithm with Stan 2.35.0 (Stan Development Team, 2024).

## **2.1 Cited literature**

- Gabry J, Cesnovar R, Johnson A, Bronder S (2024). cmdstanr: R Interface to ‘CmdStan’_. R package version 0.8.1, [https://discourse.mc-stan.org,<https://mc-stan.org/cmdstanr/](about:blank).
- McElreath, R. 2020. Statistical rethinking: A Bayesian course with examples in R and STAN. Second edition. CRC Press, London, England.
- R Core Team (2024). R: A Language and Environment for Statistical Computing. R Foundation for Statistical Computing, Vienna, Austria. [https://www.R-project.org/](https://www.r-project.org/).
- Stan Development Team. 2024. Stan Modeling Language Users Guide and Reference Manual, 2.35.0. <https://mc-stan.org>

# **3. Section ii**

## **3.1 Extracting rainfall data**

This section shows the process for choosing the time series with the highest quality data, as well as the processes needed for cleaning and preparing it to fit the model. We provide figures to highlight the available data sets.

pks <- c('readxl', 'dplyr', 'lubridate', 'ggplot2',
 'magrittr', 'cmdstanr', 'tidyr', 'forcats',
 'patchwork')

sapply(pks, FUN = function(x) library(x, character.only = T))

source('functions_mod_diagnostics.r')

stations_position <- read_xlsx('stations_location.xlsx', sheet = 1)

av_data <- as_tibble(read.csv('rainfall_published.csv', header = T, dec = ','))

av_data <-
 apply(av_data, 2, FUN =
 function(x) {
 z <- x == ''
 x[z] <- NA
 x
 })

av_data <- as_tibble(av_data)

av_data <- av_data[, c(1:2, 4:grep('ZANGUENGA', colnames(av_data)))]

av_data$not_na <-
 apply(av_data, 1, FUN =
 function(x) {
 sum(!is.na(x))
 })

plot(av_data$Year, av_data$not_na,
 ylab = 'Available stations per year (annual rainfall per station)',
 xlab = 'Year')
abline(v = c(2009, 2023), col = 'red', lty = 3, lwd = 2)


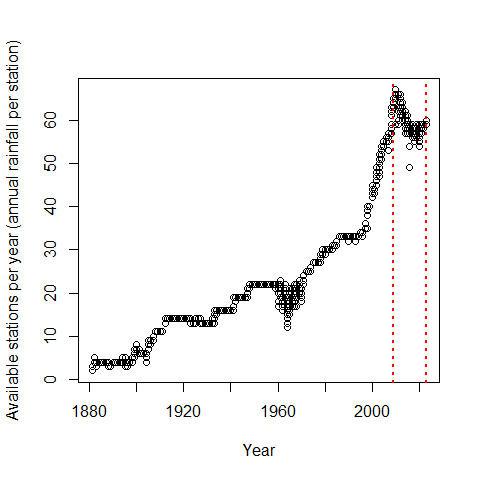


*Number of climatic stations per year with annual rainfall data at the Panama Isthmus. Vertical red dashed lines indicate the time series interval (2009 - 2023) with the higher number of stations*

The next code extracts raw rainfall data from all stations to estimate its annual rainfall.

files <- dir(paste(getwd(), '/stations_all', sep = ''))

files <- files[grepl('_ra', files)]

daily_rainfall_stations <-
 lapply(files, FUN =
 function(x) {

 message(paste('extracting from station', x))

 path <- paste(getwd(), '/stations_all/', x, sep = '')

 df <- as_tibble(read.csv(path[1], header = T))

 df <- df[df$ra >= 0, ]


 df <-
 df |>
 group_by(date) |>
 transmute(ra = sum(ra)) |>
 unique()

 df$date <- as.Date(df$date, '%d/%m/%Y')
 df$year <- year(df$date)


 df <-
 df |>
 group_by(year) |>
 transmute(ra = sum(ra)) |>
 unique()

 df$station <- x
 df

 })

Filtering data from 2009 to 2023.

daily_rainfall_stations <- do.call('rbind', daily_rainfall_stations)

daily_rainfall_stations <-
 daily_rainfall_stations[!(daily_rainfall_stations$ra > 8e3), ]

daily_rainfall_stations <-
 daily_rainfall_stations[daily_rainfall_stations$year >= 2009, ]

daily_rainfall_stations <-
 daily_rainfall_stations[daily_rainfall_stations$ra > 100, ]

daily_rainfall_stations |>
 ggplot(aes(year, ra, color = station)) +
 scale_x_continuous(breaks = 2009:2023) +
 geom_line() +
 labs(x = 'Year', y = 'Annual rainfall') +
 theme(legend.position = 'none')


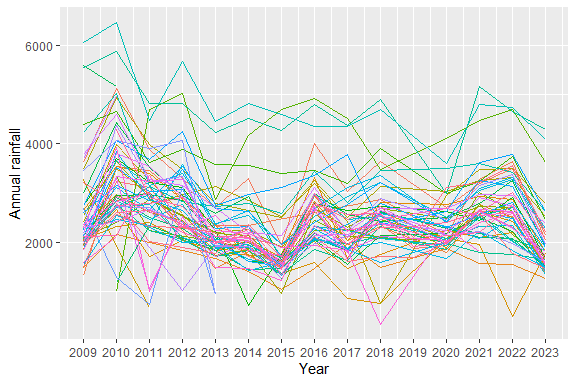


*Observed annual rainfall from the period 2009-2023 at the Panama isthmus. Colors indicate different climatic stations*

Code for standardizing character strings and filtering years lacking information.

daily_rainfall_stations$station <-
 gsub('^(.*)(_)(.*)', '\\1', daily_rainfall_stations$station)

daily_rainfall_stations$station <- tolower(daily_rainfall_stations$station)

daily_rainfall_stations <-
 split(daily_rainfall_stations, daily_rainfall_stations$year)

daily_rainfall_stations <-
 daily_rainfall_stations[names(daily_rainfall_stations) != '2019']

daily_rainfall_stations <- do.call('rbind', daily_rainfall_stations)

stations_position$nom <- stations_position$NOMBRE

stations_position$nom <-
 tolower(gsub('^(.*)(\\s)(.*)$',
 '\\1\\3',
 stations_position$nom))

stations_position$nom <-
 lapply(strsplit(stations_position$nom, ''),
 FUN =
 function(x) {
 paste(x[1], x[2], x[3], x[4], x[5], sep = '')
 }) |> unlist()

stations_position$nom[grep("ñ", stations_position$nom)] <-
 c('canoq', 'cano ')

daily_rainfall_stations$nom <-
 lapply(strsplit(daily_rainfall_stations$station, ''),
 FUN =
 function(x) {
 paste(x[1], x[2], x[3], x[4], x[5], sep = '')
 }) |> unlist()

Function for choosing specific stations:

fun_stations <-
 function(d = unique(daily_rainfall_stations[, 3:4]), i = 1) {

 str1 <- paste('^', d$nom[i], sep = '')

 station_disp <- grep(str1, stations_position$nom)
 station_disp_char <- unique(stations_position$NOMBRE[station_disp])

 message(paste('you requested the station:', d$station[i]))
 message('the available stations are:')

 if (length(station_disp_char) == 0) {
 message('The requested station is not available')
 message('See the possible stations and look if there is a match:')

 print(sort(unique(stations_position$NOMBRE)))

 } else {
 print(station_disp_char)
 }

 p1 <- readline('Do you want to continue? (y/n):')

 if (p1 == 'y') {

 station <- readline('Choose your station: ')

 df <- unique(stations_position[stations_position$NOMBRE == station, ])
 df$station <- d$station[i]
 df

 } else {
 message('Action stoped')
 }

 }

stations_position1 <-
 lapply(1:nrow(unique(daily_rainfall_stations[, 3:4])), FUN =
 function(x) {
 fun_stations(i = x)
 })

## **3.2 Model fitting**

### **3.2.1 Data preparation**

complete_stations <- unique(daily_rainfall_stations[, 3:4])

complete_stations$data_complet <- !lapply(stations_position1, is.null) |> unlist()

stations_position1 <- stations_position1[complete_stations$data_complet]

stations_position1 <-
 lapply(stations_position1, FUN =
 function(x) {

 t <-
 full_join(x[, -7],
 daily_rainfall_stations, by = 'station')

 t[!is.na(t$ID), ]

 })

Warning in full_join(x[, -7], daily_rainfall_stations, by = "station"): Detected an unexpected many-to-many relationship between `x` and `y`.
ℹ Row 1 of `x` matches multiple rows in `y`.
ℹ Row 5 of `y` matches multiple rows in `x`.
ℹ If a many-to-many relationship is expected, set `relationship =
 "many-to-many"` to silence this warning.

stations_position1 <- do.call('rbind', stations_position1)

stations_position1 <- stations_position1[, 3:9]

stations_position1$w_scaled <- as.vector(scale(stations_position1$UTM_W))
stations_position1$n_scaled <- as.vector(scale(stations_position1$UTM_N))
stations_position1$alt_scaled <- as.vector(scale(stations_position1$alt_m))
stations_position1$ra_scaled <- as.vector(scale(stations_position1$ra))

stations_position1$NOMBRE <- as.factor(stations_position1$NOMBRE)
stations_position1$name_id <- as.numeric(stations_position1$NOMBRE)
stations_position1 <-
 unique(stations_position1[order(stations_position1$name_id), ])

stations_position1$year <- as.factor(stations_position1$year)
stations_position1$year_id <- as.numeric(stations_position1$year)

chooshen_stations <- unique(stations_position1[, c("name_id", 'n_scaled', 'w_scaled')])

chooshen_stations <- chooshen_stations[-5, ]

### **3.2.2 Matrix of distance among stations**

distance <-
 lapply(seq_along(chooshen_stations$w_scaled), FUN =
 function(x) {
 W <- chooshen_stations$w_scaled[x]
 N <- chooshen_stations$n_scaled[x]
 tempX <- (W - chooshen_stations$w_scaled)^2 +
 (N - chooshen_stations$n_scaled)^2
 tempX <- sqrt(tempX)

 tibble(distance = tempX)
 })

distance <- as.matrix(do.call('cbind', distance))

### **3.2.3 Data partition for training (80%) and testing (20%) the model**

stations_position1 <-
 split(stations_position1, stations_position1$year)

dat <- stations_position1

indx_validation <-
 lapply(seq_along(stations_position1), FUN =
 function(i) {

 x <- stations_position1[[i]]
 s <- round(nrow(x) * 0.2)
 set.seed(50+i)
 sample(1:nrow(x), s)

 })

dat_validation <- # Data for validating the model
 lapply(seq_along(dat), FUN =
 function(i) {
 dat[[i]][indx_validation[[i]], ]
 })

dat_training_coords <- # data for fitting the model
 lapply(seq_along(dat), FUN =
 function(i) {
 dat[[i]][-indx_validation[[i]], ]
 })

dat_training_coords <- do.call('rbind', dat_training_coords)
colnames(dat_training_coords)

[1] "NOMBRE" "alt_m" "UTM_N" "UTM_W" "station"
 [6] "year" "ra" "w_scaled" "n_scaled" "alt_scaled"
[11] "ra_scaled" "name_id" "year_id"

dat_training_coords <- unique(dat_training_coords[, c(1, 3:4, 10, 12)])
dat_training_coords <- dat_training_coords[-4, ]
dat_training_coords <- dat_training_coords[order(dat_training_coords$name_id), ]

fun_stations <-
 function(x, y, n, testing = T) {

 tempX <- (x - dat_training_coords$UTM_W)^2 +
 (y - dat_training_coords$UTM_N)^2
 tempX <- sqrt(tempX)

 d <- dat_training_coords

 d$dist <- tempX

 d <- d[order(d$dist), ]

 if (testing) d[2:n, ]$name_id
 else d[1:n, ]$name_id
 }

dat_training <- # data for fitting the model
 lapply(seq_along(dat), FUN =
 function(i) {
 dat[[i]][-indx_validation[[i]], ]
 })

dat_training <- do.call('rbind', dat_training)

dat_training <-
 list(N = nrow(dat_training),
 N_year = max(dat_training$year_id),
 N_station = max(dat_training$name_id),
 year = dat_training$year_id,
 N_dim_dist = dim(distance)[1],
 station = dat_training$name_id,
 w_coord = dat_training$w_scaled,
 n_coord = dat_training$n_scaled,
 alt = dat_training$alt_scaled,
 rainfall = dat_training$ra_scaled,
 dist_mat = distance)

rescaled <- do.call('rbind', dat)

rescaled <- tibble(mu_ra = mean(rescaled$ra),
 sd_ra = sd(rescaled$ra),
 mu_W = mean(rescaled$UTM_W),
 sd_W = sd(rescaled$UTM_W),
 mu_N = mean(rescaled$UTM_N),
 sd_N = sd(rescaled$UTM_N),
 mu_alt = mean(rescaled$alt_m),
 sd_alt = sd(rescaled$alt_m))

### **3.2.4 Model: Stan code**

cat(file = 'predictive_model.stan',
 "
 functions {

 matrix cov_GPL2(matrix x,
 real eta,
 real rho,
 real delta) {

 int N = dims(x)[1];
 matrix[N, N] K;

 for (i in 1:(N-1)) {
 K[i, i] = eta + delta;
 for (j in (i + 1):N) {
 K[i, j] = eta * exp(-rho * square(x[i, j]));
 K[j, i] = K[i, j];
 }
 }
 K[N, N] = eta + delta;
 return K;

 }

 }

 data {

 int N;
 int N_year;
 int N_station;
 int N_dim_dist;
 array[N] int year;
 array[N] int station;
 vector[N] w_coord;
 vector[N] n_coord;
 vector[N] alt;
 vector[N] rainfall;
 matrix[N_dim_dist, N_dim_dist] dist_mat;
 }

 parameters {
 vector[N_station] z_tau;
 real<lower = 0> eta;
 real<lower = 0> rho;

 vector[N_year] z_beta_alt;
 real mu_beta_alt;
 real<lower = 0> sigma_beta_alt;

 matrix[N_year, N_year] z_alpha;
 cholesky_factor_corr[N_year] rho_alpha;
 vector<lower = 0>[N_year] sigma_alpha;
 vector[N_year] alpha_bar;

 matrix[N_year, N_year] z_beta;
 cholesky_factor_corr[N_year] rho_beta;
 vector<lower = 0>[N_year] sigma_beta;
 vector[N_year] beta_bar;

 real<lower = 0> sigma;
 }

 transformed parameters{

 vector[N_station] tau;
 matrix[N_dim_dist, N_dim_dist] sigma_tau;
 matrix[N_dim_dist, N_dim_dist] L_sigma_tau;
 sigma_tau = cov_GPL2(dist_mat, eta, rho, 0.01);
 L_sigma_tau = cholesky_decompose(sigma_tau);
 tau = L_sigma_tau * z_tau;

 vector[N_year] alpha;
 vector[N_year] betaW;
 vector[N_year] betaN;
 matrix[N_year, N_year] M_alpha;
 matrix[N_year, N_year] M_beta;
 M_alpha = (diag_pre_multiply(sigma_alpha, rho_alpha) * z_alpha)';
 M_beta = (diag_pre_multiply(sigma_beta, rho_beta) * z_beta)';
 alpha = alpha_bar[1] + M_alpha[, 1];
 betaN = alpha_bar[2] + M_alpha[, 2];
 betaW = beta_bar[1] + M_beta[, 1];

 vector[N_year] beta_alt;
 beta_alt = mu_beta_alt + z_beta_alt * sigma_beta_alt;

 }

 model {
 vector[N] mu;
 sigma ~ exponential(1);
 sigma_alpha ~ exponential(1);
 sigma_beta ~ exponential(1);
 alpha_bar ~ normal(0, 1);
 beta_bar ~ normal(0, 1);
 rho_alpha ~ lkj_corr_cholesky(2);
 rho_alpha ~ lkj_corr_cholesky(2);
 to_vector(z_alpha) ~ normal(0, 1);
 to_vector(z_beta) ~ normal(0, 1);
 z_tau ~ normal(0, 1);
 eta ~ exponential(1);
 rho ~ exponential(1);
 mu_beta_alt ~ normal(0, 1);
 z_beta_alt ~ normal(0, 1);
 sigma_beta_alt ~ exponential(1);

 for (i in 1:N) {
 mu[i] = alpha[year[i]] + tau[station[i]] +
 betaN[year[i]]*n_coord[i] +
 betaW[year[i]]*w_coord[i] +
 beta_alt[year[i]]*alt[i];
 }

 rainfall ~ student_t(2, mu, sigma);

 }

 generated quantities {

 vector[N] mu;
 array[N] real ppcheck;

 for (i in 1:N) {
 mu[i] = alpha[year[i]] + tau[station[i]] +
 betaN[year[i]]*n_coord[i] +
 betaW[year[i]]*w_coord[i] +
 beta_alt[year[i]]*alt[i];
 }

 ppcheck = student_t_rng(2, mu, sigma);

 }
 ")

### **3.2.5 Running Hamiltonian MCMC algorithm**

pred_mod <-
 fit$sample(
 data = dat_training,
 iter_warmup = 500,
 iter_sampling = 3e3,
 chains = 3,
 parallel_chains = 3,
 thin = 3,
 refresh = 500,
 seed = 123
 )

### **3.2.6 Model diagnostics**

ppcheck <- pred_mod$draws('ppcheck', format = 'matrix')

plot(density(dat_training$rainfall), main = '', xlab = 'Annual rainfall (z-scores)')
for (i in 1:100) lines(density(ppcheck[i, ]), lwd = 0.1)
lines(density(dat_training$rainfall), col = 'red')


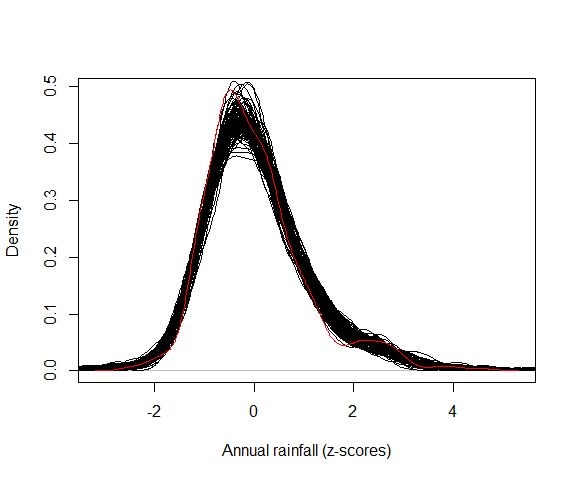


*Posterior predictive checks. Black lines indicate a posterior prediction of the training data. The red line shows the observed values*

summ <- pred_mod$summary(c('alpha', 'tau', 'betaN', 'betaW', 'beta_alt'))
mod_diagnostics(pred_mod, summ)


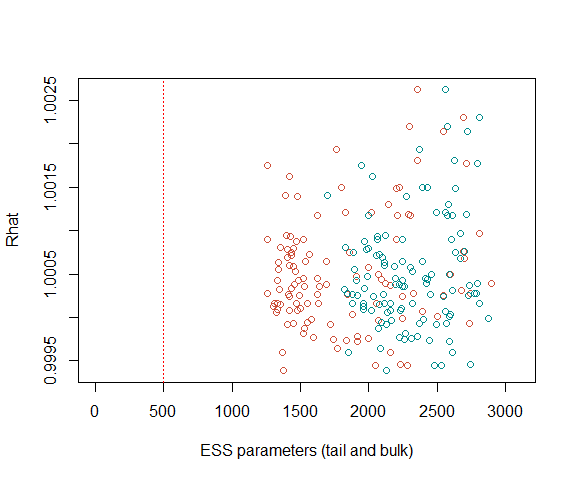


*Bulk and tail effective sampling size (ess) of model’s parameters. All ess values are above 1000, indicating that all parameters had enough independent random samples. Rhat values below 1.1 indicate that the chains of all parameters converged to similar space*

## **3.3 Testing prediction performance**

Below, we simulate random partitions of the data in training (80%) and testing (20%) sets to fit the model. The iteration follows the next steps: (i) random partition of the data and formatting, (ii) running the MCMC algorithm, and (ii) calculating the accuracy of annual rainfall estimation.

t1 <- Sys.time()
simulations <-
 lapply(1:10, FUN =
 function(i) {

 # ===== data preparation

 indx_validation <-
 lapply(seq_along(stations_position1), FUN =
 function(j) {

 x <- stations_position1[[j]]
 s <- round(nrow(x) * 0.2)
 set.seed(50+i)
 sample(1:nrow(x), s)

 })

 dat_validation <- # Data for validating the model
 lapply(seq_along(dat), FUN =
 function(k) {
 dat[[k]][indx_validation[[k]], ]
 })

 dat_training_coords <- # data for fitting the model
 lapply(seq_along(dat), FUN =
 function(l) {
 dat[[l]][-indx_validation[[l]], ]
 })

 dat_training_coords <- do.call('rbind', dat_training_coords)

 dat_training_coords <- unique(dat_training_coords[, c(1, 3:4, 10)])
 dat_training_coords <- dat_training_coords[-4, ]
 dat_training_coords <- dat_training_coords[order(dat_training_coords$name_id), ]

 fun_stations <-
 function(x, y, n, testing = T) {

 tempX <- (x - dat_training_coords$UTM_W)^2 +
 (y - dat_training_coords$UTM_N)^2
 tempX <- sqrt(tempX)

 d <- dat_training_coords

 d$dist <- tempX

 d <- d[order(d$dist), ]

 if (testing) d[2:n, ]$name_id
 else d[1:n, ]$name_id
 }

 dat_training <- # data for fitting the model
 lapply(seq_along(dat), FUN =
 function(m) {
 dat[[m]][-indx_validation[[m]], ]
 })

 dat_training <- do.call('rbind', dat_training)

 dat_ <-
 list(N = nrow(dat_training),
 N_year = max(dat_training$year_id),
 N_station = max(dat_training$name_id),
 year = dat_training$year_id,
 N_dim_dist = dim(distance)[1],
 station = dat_training$name_id,
 w_coord = dat_training$w_scaled,
 n_coord = dat_training$n_scaled,
 alt = dat_training$alt_scaled,
 rainfall = dat_training$ra_scaled,
 dist_mat = distance)

 # ====== model fitting

 pred_mod <-
 fit$sample(
 data = dat_,
 iter_warmup = 500,
 iter_sampling = 3e3,
 chains = 3,
 parallel_chains = 3,
 thin = 3,
 refresh = 500,
 seed = 123
 )

 # ========= accuracy of annual rainfall estimation

 post <- pred_mod$draws(c('alpha', 'tau', 'betaN', 'betaW', 'beta_alt'),
 format = 'df')

 post <-
 list(alpha = post[, grep('alpha', colnames(post))],
 tau = post[, grep('tau', colnames(post))],
 betaN = post[, grep('betaN', colnames(post))],
 betaW = post[, grep('betaW', colnames(post))],
 beta_alt = post[, grep('beta_alt', colnames(post))])


 pred_validation <-
 lapply(seq_along(dat_validation), FUN =
 function(zz) {

 n <- dat_validation[[zz]]

 d <-
 lapply(1:nrow(n), FUN =
 function(x) {

 alt <- n$alt_scaled[x]
 north <- n$n_scaled[x]
 west <- n$w_scaled[x]
 X <- n$UTM_W[x]
 Y <- n$UTM_N[x]

 stations <-
 fun_stations(X, Y, n = 4, F)

 p <-
 with(post,
 {
 alpha[, zz, drop = T] +
 apply(tau[, stations], 1, mean) +
 betaN[, zz, drop = T]*north +
 betaW[, zz, drop = T]*west +
 beta_alt[, zz, drop = T]*alt
 })

 p <- rescaled$mu_ra + p * rescaled$sd_ra
 res_i <-
 rescaled$mu_ra + n$ra_scaled[x] * rescaled$sd_ra
 res_north <-
 rescaled$mu_N + north * rescaled$sd_N
 res_west <-
 rescaled$mu_W + west * rescaled$sd_W

 tibble(indx_dat = x,
 data = c(res_i, mean(p)),
 type_data = c('obs', 'pred'),
 diff = mean(p) - res_i,
 perc_error =
 (abs(mean(p) - res_i) * 100) / res_i,
 li_pred = quantile(p, 0.025),
 ls_pred = quantile(p, 0.975),
 year = n$year[x],
 north_coord = res_north,
 west_coord = res_west)

 })

 do.call('rbind', d)

 })

 pred_validation <- do.call('rbind', pred_validation)

 message(paste('Finishing simulation', i))

 pred_validation$sim <- paste('sim', i, sep = '_')
 pred_validation

 })
Sys.time() - t1

do.call('rbind', simulations) |>
 select(-data, -type_data) |>
 unique() |>
 group_by(year, sim) |>
 transmute(perc = median(perc_error),
 year = as.numeric(as.character(year))) |>
 unique() |>
 ggplot() +
 geom_point(aes(year, perc, color = as.factor(sim))) +
 geom_line(aes(year, perc, color = as.factor(sim))) +
 scale_x_continuous(breaks = 2009:2023) +
 scale_y_continuous(breaks = seq(0, 45, by = 5)) +
 labs(x = 'year', y = 'Percentage of the error\n on predicted rainfall') +
 theme(legend.position = 'none')


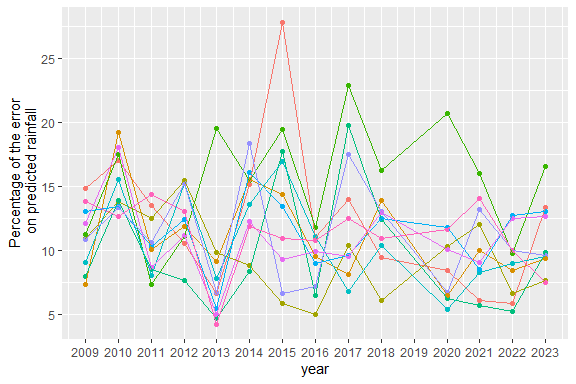


*Predicted annual rainfall per year. Colored lines indicate different rainfall stations.*

Average and SD of error percentage for the prediction of annual rainfall per station.

pred_accuracy <-
 do.call('rbind', simulations) |>
 select(-data, -type_data) |>
 unique() |>
 group_by(year, sim) |>
 transmute(perc = median(perc_error),
 year = as.numeric(as.character(year))) |>
 unique()

mean(100 - pred_accuracy$perc)

[1] 88.62935

sd(100 - pred_accuracy$perc)

[1] 4.055439

After estimating the average error, we use one of the models to predict annual rainfall at our sampling sites.

post <- pred_mod$draws(c('alpha', 'tau', 'betaN', 'betaW', 'beta_alt'),
 format = 'df')

post <-
 list(alpha = post[, grep('alpha', colnames(post))],
 tau = post[, grep('tau', colnames(post))],
 betaN = post[, grep('betaN', colnames(post))],
 betaW = post[, grep('betaW', colnames(post))],
 beta_alt = post[, grep('beta_alt', colnames(post))])

Warning: Dropping 'draws_df' class as required metadata was removed.
Warning: Dropping 'draws_df' class as required metadata was removed.
Warning: Dropping 'draws_df' class as required metadata was removed.
Warning: Dropping 'draws_df' class as required metadata was removed.
Warning: Dropping 'draws_df' class as required metadata was removed.

pred_validation <-
 lapply(seq_along(dat_validation), FUN =
 function(j) {

 i <- dat_validation[[j]]

 d <-
 lapply(1:nrow(i), FUN =
 function(x) {

 alt <- i$alt_scaled[x]
 north <- i$n_scaled[x]
 west <- i$w_scaled[x]
 X <- i$UTM_W[x]
 Y <- i$UTM_N[x]

 stations <-
 fun_stations(X, Y, n = 4)

 p <-
 with(post,
 {
 alpha[, j, drop = T] +
 apply(tau[, stations], 1, mean) +
 betaN[, j, drop = T]*north +
 betaW[, j, drop = T]*west +
 beta_alt[, j, drop = T]*alt
 })

 p <- rescaled$mu_ra + p * rescaled$sd_ra
 res_i <- rescaled$mu_ra + i$ra_scaled[x] * rescaled$sd_ra
 res_north <- rescaled$mu_N + north * rescaled$sd_N
 res_west <- rescaled$mu_W + west * rescaled$sd_W

 tibble(indx_dat = x,
 data = c(res_i, mean(p)),
 type_data = c('obs', 'pred'),
 diff = mean(p) - res_i,
 perc_error = (abs(mean(p) - res_i) * 100) / res_i,
 li_pred = quantile(p, 0.025),
 ls_pred = quantile(p, 0.975),
 year = i$year[x],
 north_coord = res_north,
 west_coord = res_west)

 })

 d <- do.call('rbind', d)
 as_tibble(d)

 })

pred_validation <- do.call('rbind', pred_validation)

pred_validation |>
 ggplot() +
 geom_boxplot(aes(type_data, data)) +
 geom_jitter(aes(type_data, data), width = 0.1, size = 0.25) +
 facet_wrap(~year, scales = 'free') +
 labs(x = 'Data', y = 'Annual rainfall (mm)')


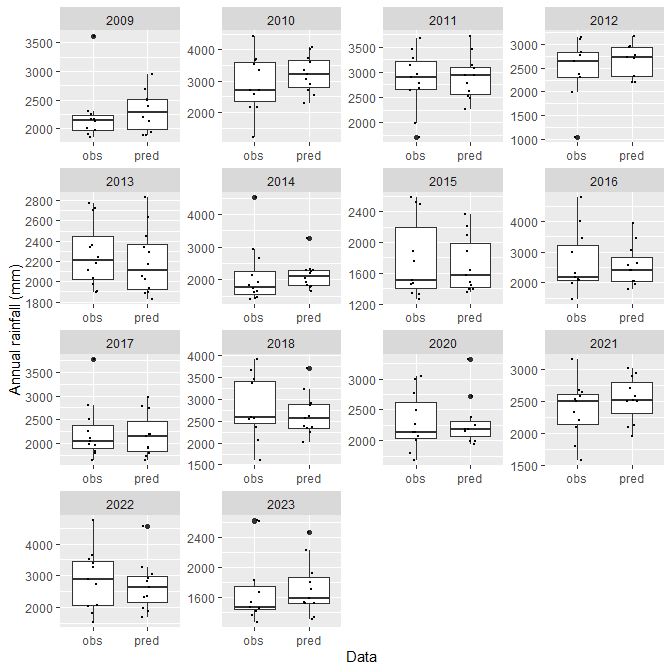


*Predicted vs observed annual rainfall values. Single dotes show the data of each station*

pred_validation |>
 ggplot() +
 geom_density(aes(data, y = after_stat(..scaled..), color = type_data)) +
 facet_wrap(~year, scales = 'free') +
 labs(y = 'Density', x = 'Annual rainfall (mm)')


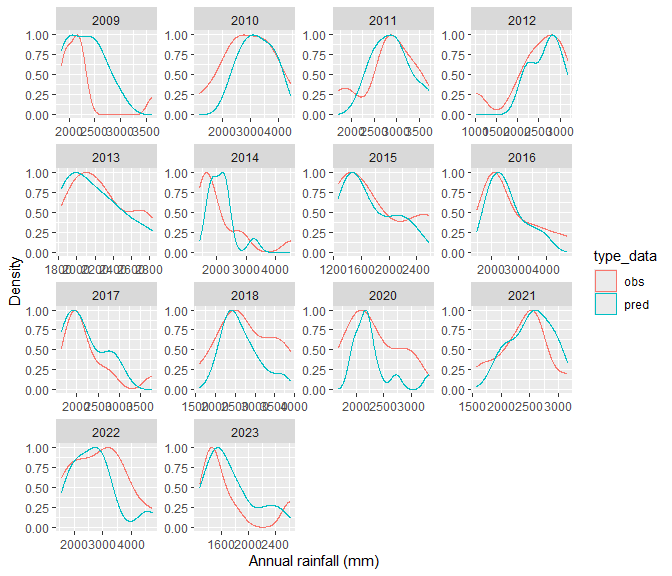


*Predicted (pred) vs observed (obs) annual rainfall values*

pred_validation |>
 select(-data, -type_data) |>
 unique() |>
 group_by(year) |>
 transmute(diff = median(diff)) |>
 unique() |>
 ggplot(aes(as.factor(year), diff)) +
 geom_point() +
 geom_hline(yintercept = 0, linetype = 3) +
 labs(x = 'Year', y = 'Annual rainfall (mm)')


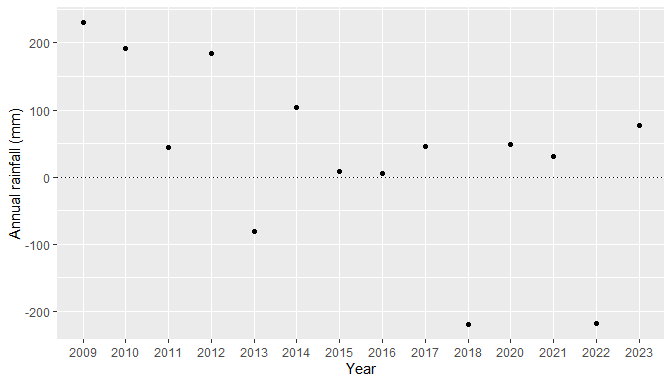


*Average over or underestimation of annual rainfall*

knitr::kable(
 pred_validation |>
 select(-data, -type_data) |>
 unique() |>
 group_by(year) |>
 transmute(diff = abs(median(diff))) |>
 ungroup() |>
 transmute(mu_error = mean(diff),
 sd_error = sd(diff)) |>
 unique() |>
 data.frame()
)

*Total error (average and SD) on annual rainfall prediction (mm)*

| mu_error | sd_error |
| --- | --- |
| 104.2591 | 80.58325 |

pred_map <-
 pred_validation |>
 select(-data, -type_data) |>
 unique()

map <- do.call('rbind', dat)
map_trainin <- do.call('rbind', dat_validation)

pred_map$check <- pred_map$perc_error < 25
pred_map$check1 <- pred_map$perc_error > 25 & pred_map$perc_error < 30
pred_map$check3 <- pred_map$perc_error > 30

par(mfrow = c(1, 2), mar = c(4, 4, 1, 1))
pred_map %$% plot(north_coord, perc_error,
 xlab = 'UTM N', ylab = 'Percentage of error')
pred_map[pred_map$check,] %$%
 points(north_coord, perc_error,
 col = 'tan1', pch = 16)
pred_map[pred_map$check1,] %$%
 points(north_coord, perc_error,
 col = 'green', pch = 16)
pred_map[pred_map$check3,] %$%
 points(north_coord, perc_error,
 col = 'yellow', pch = 16)
pred_map[pred_map$perc_error > 100,] %$%
 points(north_coord, perc_error,
 col = 'red', pch = 16)

pred_map %$% plot(west_coord, perc_error,
 xlab = 'UTM W', ylab = 'Percentage of error')
pred_map[pred_map$check,] %$%
 points(west_coord, perc_error,
 col = 'tan1', pch = 16)
pred_map[pred_map$check1,] %$%
 points(west_coord, perc_error,
 col = 'green', pch = 16)
pred_map[pred_map$check3,] %$%
 points(west_coord, perc_error,
 col = 'yellow', pch = 16)
pred_map[pred_map$perc_error > 100,] %$%
 points(west_coord, perc_error,
 col = 'red', pch = 16)


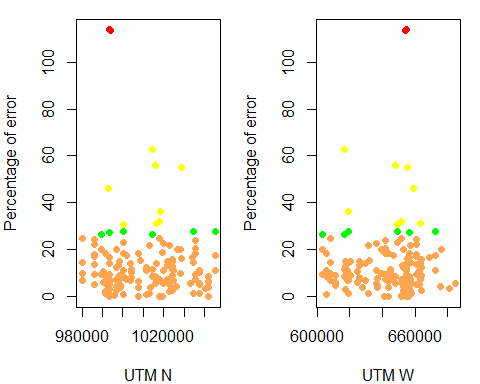


*Error on annual rainfall prediction (percentage). Single points indicate data per station/year*

## **3.4 Predicting annual rainfall**

Here, we use the actual UTM coordinates and altitude of our sampling sites to estimate annual rainfall.

to_pred <- as_tibble(read.csv('sites_with_elevation.csv',
 dec = '.', header = T))

to_pred$z_W <- (to_pred$utm_W - rescaled$mu_W) / rescaled$sd_W
to_pred$z_N <- (to_pred$utm_N - rescaled$mu_N) / rescaled$sd_N
to_pred$z_alt <- (to_pred$elevation - rescaled$mu_alt) /rescaled$sd_alt

fun_stations(to_pred$utm_W[1], to_pred$utm_N[1], n = 3, testing = F)

[1] 33 21 25

years_codes <- unique(do.call('rbind', dat_validation)[, c('year', 'year_id')])

predicted_rainfall <-
 lapply(seq_along(to_pred$Site_name), FUN =
 function(j) {

 w <- to_pred$z_W[j]
 n <- to_pred$z_N[j]
 alt <- to_pred$z_alt[j]

 d <-
 lapply(seq_along(post$alpha), FUN =
 function(x) {

 stations <-
 fun_stations(to_pred$utm_W[j],
 to_pred$utm_N[j],
 n = 3, testing = F)

 p <-
 with(post,
 {
 alpha[, x, drop = T] +
 apply(tau[, stations], 1, mean) +
 betaN[, x, drop = T]*n +
 betaW[, x, drop = T]*w +
 beta_alt[, x, drop = T]*alt
 })

 p <- rescaled$mu_ra + p * rescaled$sd_ra
 utm_N <- rescaled$mu_N + n * rescaled$sd_N
 utm_W <- rescaled$mu_N + n * rescaled$sd_N

 tibble(site = to_pred$Site_name[j],
 year = years_codes$year[x],
 annual_rainfall = mean(p),
 CI95_l = quantile(p, 0.025),
 CI95_u = quantile(p, 0.975),
 utm_N = to_pred$utm_N[j],
 utm_W = to_pred$utm_W[j],
 w_z = w,
 n_z = n,
 altitude = to_pred$elevation[j])

 })

 do.call('rbind', d)

 })

predicted_rainfall <- do.call('rbind', predicted_rainfall)

jitter <- rnorm(nrow(predicted_rainfall), 15000, 2500)

p1 <-
 predicted_rainfall |>
 ggplot() +
 geom_point(aes(utm_N + jitter, annual_rainfall,
 color = year), alpha = 0.5) +
 geom_errorbar(aes(utm_N + jitter,
 ymin = CI95_l,
 ymax = CI95_u,
 color = year), alpha = 0.5) +
 geom_smooth(aes(utm_N + jitter, annual_rainfall,
 color = year), se = F, method = 'lm',
 linewidth = 0.3) +
 labs(y = 'Annual rainfall', x = 'UTM N')


p2 <-
 predicted_rainfall |>
 ggplot() +
 geom_point(aes(utm_W + jitter, annual_rainfall,
 color = year), alpha = 0.5) +
 geom_errorbar(aes(utm_W + jitter,
 ymin = CI95_l,
 ymax = CI95_u,
 color = year), alpha = 0.5) +
 geom_smooth(aes(utm_W + jitter, annual_rainfall,
 color = year), se = F, method = 'lm',
 linewidth = 0.3) +
 labs(y = 'Annual rainfall', x = 'UTM W') +
 theme(legend.position = 'none')

p1 + p2 + plot_layout(ncol = 1)

`geom_smooth()` using formula = 'y ~ x'
`geom_smooth()` using formula = 'y ~ x'


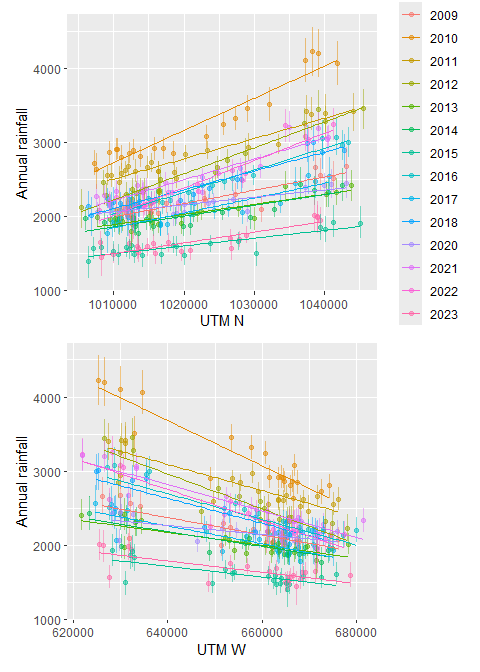


*Predicted annual rainfall at each sampling site during the years 2009-2023. Error bars indicate 95% credibility intervals for the prediction of each single point*

sessionInfo()

R version 4.4.0 (2024-04-24 ucrt)
Platform: x86_64-w64-mingw32/x64
Running under: Windows 11 x64 (build 26100)

Matrix products: default


locale:
[1] LC_COLLATE=Spanish_Latin America.utf8
[2] LC_CTYPE=Spanish_Latin America.utf8
[3] LC_MONETARY=Spanish_Latin America.utf8
[4] LC_NUMERIC=C
[5] LC_TIME=Spanish_Latin America.utf8

time zone: America/Sao_Paulo
tzcode source: internal

attached base packages:
[1] stats graphics grDevices utils datasets methods base

other attached packages:
[1] patchwork_1.2.0 forcats_1.0.0 tidyr_1.3.1 cmdstanr_0.8.1
[5] magrittr_2.0.3 ggplot2_3.5.1 lubridate_1.9.3 dplyr_1.1.4
[9] readxl_1.4.3

loaded via a namespace (and not attached):
 [1] tensorA_0.36.2.1 utf8_1.2.4 generics_0.1.3
 [4] lattice_0.22-6 digest_0.6.36 evaluate_0.24.0
 [7] grid_4.4.0 timechange_0.3.0 fastmap_1.2.0
[10] Matrix_1.7-0 cellranger_1.1.0 jsonlite_1.8.8
[13] processx_3.8.4 backports_1.5.0 ps_1.7.7
[16] mgcv_1.9-1 purrr_1.0.2 fansi_1.0.6
[19] scales_1.3.0 abind_1.4-5 cli_3.6.3
[22] rlang_1.1.4 splines_4.4.0 munsell_0.5.1
[25] withr_3.0.0 yaml_2.3.9 tools_4.4.0
[28] checkmate_2.3.1 colorspace_2.1-0 vctrs_0.6.5
[31] posterior_1.6.0 R6_2.5.1 matrixStats_1.3.0
[34] lifecycle_1.0.4 pkgconfig_2.0.3 pillar_1.9.0
[37] gtable_0.3.5 glue_1.7.0 xfun_0.45
[40] tibble_3.2.1 tidyselect_1.2.1 rstudioapi_0.16.0
[43] knitr_1.48 farver_2.1.2 nlme_3.1-164
[46] htmltools_0.5.8.1 rmarkdown_2.27 labeling_0.4.3
[49] compiler_4.4.0 distributional_0.4.0
